# Performance, Rumen Microbial Community and Immune Status of Goat Kids Fed *Leucaena leucocephala* Post-weaning as Affected by Prenatal and Early Life Nutritional Interventions

**DOI:** 10.3389/fmicb.2021.769438

**Published:** 2022-02-16

**Authors:** Einar Artiles-Ortega, Orelvis Portal, Jeyamalar Jeyanathan, Beydis Reguera-Barreto, Pedro Yoelvys de la Fé-Rodríguez, Raciel Lima-Orozco, Veerle Fievez

**Affiliations:** ^1^Laboratory for Animal Nutrition and Animal Product Quality, Department of Animal Sciences and Aquatic Ecology, Faculty of Bioscience Engineering, Ghent University, Ghent, Belgium; ^2^Departamento de Medicina Veterinaria y Zootecnia, Facultad de Ciencias Agropecuarias, Universidad Central “Marta Abreu” de Las Villas, Santa Clara, Cuba; ^3^Departamento de Biología, Facultad de Ciencias Agropecuarias, Universidad Central “Marta Abreu” de Las Villas, Santa Clara, Cuba; ^4^Centro de Investigaciones Agropecuarias, Facultad de Ciencias Agropecuarias, Universidad Central “Marta Abreu” de Las Villas, Santa Clara, Cuba

**Keywords:** goat kids, early life intervention, bacterial community, immune status, performance, digestibility

## Abstract

*Leucaena leucocephala* represents a local protein source in tropical ruminant diets. However, its full exploitation is impaired by mimosine, unless it is degraded by the rumen microbial community. Recently, the ruminal bacterial communities of newborns were persistently modified through prenatal or postnatal dietary interventions. Such early-life interventions might enhance adaptation of ruminants to *Leucaena leucocephala*, which was investigated using a 2 × 2 factorial design trial that tested both supplementation of *L. leucocephala* in the late pregnancy diet of goat does, and supplementation of live yeast to their newborns. The composition of ruminal bacteria, immune status, as well as organic matter digestibility (OMD) and performance of kids were studied during and after the intervention. Ten pregnant goats were divided into two groups: the D+ and D– groups, which either received or did not receive 30 g of *L. leucocephala* forage meal during the last 7 ± 0.5 weeks of gestation. Twins from each goat were divided into the K+ and K– group (supplemented with or without 0.2 g/d of live yeast from day 3 until weaning at 8 weeks). Rumen samples were collected from 4-, 8-, 14-, and 20-weeks old kids to assess the bacterial community, while immune parameters (white blood cells, immunoglobulin M and G, and chitotriosidase activity) were measured in blood and saliva sampled at 4-, 8-, and 20-weeks. We found a stimulatory effect of the prenatal exposure on the post-weaning dry matter intake of the *L. leucocephala* supplemented diet, resulting in a higher daily gain and final body weight at 20 weeks in the D+ *vs*. D– group (406 *vs*. 370 g DM/d, 85.4 vs. 78.6 g/d, and 15.2 vs. 13.8 kg, respectively). Moreover, *Ruminococcus* represented a greater proportion of the rumen bacterial community of the D+ vs. D– kids (5.1 vs. 1.6%). Differences in the immune status were relatively small and not thought to be a driving factor of differences in animal performance. Furthermore, postnatal supplementation of live yeast favored maturation of the rumen bacterial community (i.e., greater abundance of Bacteroidetes, in particular *Prevotella*, and reduced abundance of Firmicutes) and protozoa colonization. Concomitantly, OMD was enhanced post-weaning, suggesting effects of the early-life intervention persisted and could have affected animal performance.

## Introduction

Due to the increasing demand for animal-derived food and the restricted availability of good quality forage and concentrates, there is a need to find cheap and readily available alternative feed sources to support livestock production in tropical countries (Kim et al., 2019). Protein-rich leaves from legume trees are such feed resources (Aye and Adegun, 2013) with *Leucaena leucocephala* as one of the highly productive, palatable, and most widely used legumes in tropical agropastoral systems (Vega et al., 2016; Ahmed et al., 2018). Nevertheless, *L. leucocephala* contains toxic secondary metabolites, i.e., L-mimosine [(*S*)-α-Amino-β-[1-(3-hydroxy-4-oxopyridine)] propionic acid] and its digestive intermediates (isomers of hydroxypyridone; 2,3 and 3,4-DHP). The toxicity of these plant secondary metabolites could be alleviated through degradation by rumen microbes (Akingbade et al., 2001; Angarita et al., 2015). Indeed, in several studies, performed in tropical countries, the rumen microbial community of ruminants fed *L. leucocephala* contained some DHP degrading bacteria such as *Synergistes jonesii* (Allison et al., 1990), *Streptococcus lutetiensis*, *Clostridium butyricum*, and *Lactobacillus vitulinus* (Dominguez-Bello and Stewart, 1991; Derakhshani et al., 2016). Recently, own research (unpublished data) also showed that *in vitro* degradation of *L. leucocephala* forage meal as well as L-mimosine itself was highly influenced by the origin of the inoculum and was more extensive with inoculum from Cuban goats compared to Belgian sheep.

The transinoculation of a DHP-degrading rumen inoculum from *Leucaena*-adapted animals to non-adapted animals contributed to the detoxification of L-mimosine and its intermediates in non-adapted animals (Jones and Megarrity, 1986; Akingbade et al., 2001). However, in other research transinoculation did not show any effect on the detoxification of L-mimosine (Vaithiyanathan et al., 2005) or the detoxification effect was lost after a relatively short period with *L. leucocephala*-free diets (Graham et al., 2013). This may be due to the fact that the rumen microbiome of adult ruminants is difficult to manipulate due to the resistance of the indigenous microflora against the colonization of foreign bacterial strains (Weimer et al., 2010). In contrast, the proliferating microbial community in the rumen of young ruminants seems more moldable and several studies showed short and medium-term persistency of early life microbial manipulation (Yanez-Ruiz et al., 2010; Belanche et al., 2020). In this respect, active yeasts, used as feed additives in young ruminants, improved rumen microbial activity and particularly stimulated the growth and activity of fiber-degrading bacteria (Chaucheyras-Durand et al., 2008). Indeed, yeast supplementation as probiotic was shown to create favorable conditions allowing the earlier establishment of cellulolytic bacteria and ciliate protozoa in the rumen of newborn lambs (Chaucheyras-Durand and Fonty, 2002).

Moreover, the early-life interest has been extended to prenatal modulation of the microbial community through maternal feeding during the gestation period (Faubladier et al., 2013; Codagnone et al., 2019; Xiong et al., 2019). In ruminants, only a few studies investigated the effect of a prenatal treatment on the developing microbiome of the offspring (De Barbieri et al., 2015a,b). Nevertheless in these studies the offspring was raised with their mother during the lactation period, which compromised the separation of the prenatal effects from the mother’s influence during lactation. In addition, enhanced post-weaning intake of *Chromolaena odorata* by goat kids has been related to an *in utero* exposure period with maternal ingestion of *C. odorata* (Hai et al., 2013).

In the current study, we hypothesized that the use of *L. leucocephala*, fed after weaning, would be optimized by either or both a prenatal and postnatal treatment. The prenatal treatment included the dietary supply of *L. leucocephala* to mother goats during late pregnancy whereas live yeast supplementation until weaning was tested as postnatal treatment. The objective of this study was to assess their effects on feed intake and growth of the kids as well as on the composition of the ruminal bacterial community and their immune status during their development from 4 to 20 weeks. We also determined apparent digestibility at the age of 20 weeks.

## Materials and Methods

The experiment was in accordance with the recommendations of the Ethical Committee of the Faculty of Veterinary Medicine, Ghent University, Belgium (approval number EC2015/12) for a similar experiment conducted at the Laboratory of Animal Nutrition and Animal Product Quality of Ghent University. The current experiment was conducted in the Laboratory of Animal Nutrition and the Veterinary Clinic, Facultad de Ciencias Agropecuarias at Universidad Central “Marta Abreu” de Las Villas (UCLV), Cuba.

### Animals, Treatments and Experimental Design

#### Prenatal Treatment

**FIGURE 1:**
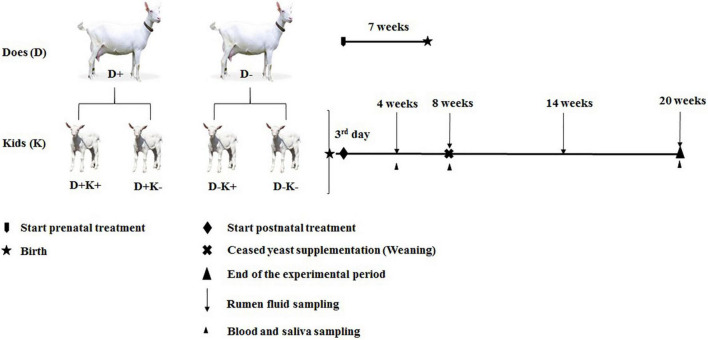
Experimental design and sampling and data collection schedule of goat kids. D+, *L. leucocephala* [LL] supplemented group of does; D–, control group of does; K+, kids treated with yeast; K–, untreated kids. Four experimental groups; D+K+, yeast supplemented kids from LL supplemented group of does; D–K+, yeast supplemented kids from Control group of does; D+K–, non-supplemented kids from LL supplemented group of does; and D–K–, non supplemented kids from Control group of does.

Ten pregnant goats (35 ± 5 kg BW) with twin pregnancies were selected from a commercial farm (total of 60 goats), where does conceived by natural mating. Twin pregnancies were identified ultrasonically 2 months after mating. The ten goats were randomly allocated to two experimental groups (D+: *L. leucocephala* [LL] supplemented group, and D–: Control group) during the last 7 ± 0.5 weeks of gestation (Figure 1). Each group was housed in separate stables with individual pens (1.0 × 1.5 m; width × length with *ad libitum* access to water). Despite their individual housing, goats had visual contact. Feed was offered twice a day, with diets consisting of 2.0 kg DM/head/day of star grass (*Cynodon nlemfuensis*) hay, and 0.369 ± 0.03 kg DM/head/day of concentrate (Supplementary Table S1). The forage meal of *L. leucocephala* was offered only to the D+ group.

The *L. leucocephala* cv. Cunningham used to prepare the forage meal was grown on the dairy farm “Tres Caminos” (22°25′22.9″ N, 80°03′13″ W) at the “Desembarco del Granma” cooperative, located in the Santa Clara municipality, central region of Cuba. The average precipitation, temperature and relative humidity during the cropping period were 47.5 ± 42.13 mm, 26.3 ± 2.02°C and 82.5 ± 4.19%, respectively. The legume forage was harvested freshly from young branches around noon, at the end of the rainy season (between the 3rd and 27th October 2018). The material selected for this research (a pool of leaves without branches) was dried in a furnace at 65°C for three days, then chopped to a particle size of 0.1 cm and stored in a dry area until use.

#### Postnatal Treatment

After birth, kids (twins) were separated from their mother immediately after the first intake of colostrum (first day), and were divided into two groups which were provided with milk replacer (125 g/L, Kalvowin, Polmass S.A., Poland [Supplementary Table S2]) twice a day until weaning (8 weeks of age). All kids had *ad libitum* access to water. One group of kids received 0.2 g of yeast (Yea Sac^®^ I-1026 [2 × 10^9^ CFU/g live *Saccharomyces cerevisiae*], Alltech, Deinze, Belgium) per head from day 3 until weaning [K+] and the other group did not receive yeast [K–]. This resulted in four experimental groups: D+K+, D+K–, D–K+ and D–K– (*n* = 5) as illustrated in Figure 1. The determination of the number of goat kids required for this experiment was based on former experiments studying persistency of early life treatments and a sample size per treatment of 4 (e.g., Wang et al., 2017), 5 (e.g., Hai et al., 2013; Wang X. et al., 2019), 8 (e.g., Abecia et al., 2018; Zhang et al., 2019), up to 10 animals (e.g., Debruyne et al., 2018). To determine the minimum sample size, a power analysis was performed using GPower version 3.1.9.7 (Supplementary Table S3). Finally, a sample size of 4–7 per treatment group was calculated to be required for a power of 80% and a type I error α of 0.05. The yeast product (small dry pellets exclusively containing yeast cells) was suspended in 10 mL of saline solution (LABIOFAM SA, Cuba) and was introduced to the rumen of the K+ kids daily before milk feeding using an esophageal tube connected to a syringe (Chaucheyras-Durand and Fonty, 2002). One kid from each of the D–K+ and D–K– groups died during the first 4 weeks of life (esophageal groove dysfunction and ruminal bloat). These deaths were not related to the experimental conditions or treatments. Furthermore, data from one kid of the D–K– group at 4-weeks of age were removed due to clinical signs of digestive disorders around the time of sampling. These problems lasted shortly and the kid recovered well afterward. Accordingly, data of the later time points were maintained in the dataset.

From day 22 until weaning (8 weeks), kids were offered star grass hay *ad libitum* and a Lacto pre-starter (50–200 g/kid; RALTEC TC-01, Serveram S.L., Barcelona, Spain [Supplementary Table S2]). After weaning, all kids received star grass hay [2/3 of the offered dietary DM] and forage meal of *L. leucocephala* [supplying 30% of the CP requirements] mixed with commercial concentrate. Feed was provided twice a day (at 8:00 and 17:00) according to the kids’ nutritive requirements (Kearl, 1982). Adjustments were made biweekly, after determining body weight before the morning feeding. Feed refusals was collected just prior to the distribution of the next feeding. The refused feed were collected and dried in an oven at 65°C for 72 h, pooled by animal/day, ground and sieved through a 1 mm screen, and stored until analysis. Average daily gain was calculated by the difference between the final and initial body weight divided by the number of days in the corresponding period. Dry matter intake (DMI) was measured daily by weighing the feed offered and refused, and the feed conversion efficiency (FCE) was calculated by the feed intake divided by the body weight gain in the same period.

#### Sampling of Ruminal Content, Saliva, Blood, and Colostrum

Ruminal contents were sampled at 4, 8, 14, and 20 weeks of age before the morning feeding, using an esophageal tube. The collected rumen fluid (2 mL) was immediately snap frozen in liquid nitrogen and stored at −80°C for microbial analysis. Saliva and blood were sampled before the morning feeding at 4, 8, and 20 weeks of age (Figure 1). Saliva was collected immediately before blood sampling by using a forcep and a small piece of absorbing sponge. The sponge was held into the kids’ mouth for 1 min to stimulate saliva production. Saliva was removed from the sponge by squeezing into a 10 mL pipet tip placed into the collection tube, and aliquots of approximately 100 μL were stored at −20°C. Blood samples of does (at kidding) and their kids (at 4, 8, and 20 weeks of age) were taken from jugular vein puncture in 20 mL assay tubes with and without anticoagulant (10% EDTA; 100 μL/mL of blood; Sigma-Aldrich, Diegem, Belgium). White blood cells (WBC) were counted using the Neubauer chamber under a binocular microscope (NOVEL, NOV-XSZ-107T, Beijing, China). Afterward, the leftover blood was centrifuged at 1500 × *g* for 5 min at room temperature to obtain plasma. Blood samples without anticoagulant (10 mL) were stored overnight at 4°C and centrifuged at 1500 × *g* for 10 min at room temperature to obtain serum. Serum aliquots of 2 mL were stored at −20°C. Colostrum (20 mL) was collected during the first hour after delivery and stored at −20°C. Due to the high viscosity of colostrum, removal of casein was required prior to the immunoglobulin determination by ELISA (Alves et al., 2015). For this, frozen samples of colostrum were thawed slowly in an ice bath and centrifuged at 490 × *g* for 1 h at 4°C to precipitate casein. The supernatant was diluted in a 0.15 M NaCl solution (pH 4.6) up to its original volume and kept overnight at 4°C. Then, the samples were centrifuged at 11,000 × *g* for 15 min at 4°C, and the supernatant (containing antibodies) was aliquoted and stored at −20°C in 2 mL polyethylene tubes.

### Bacterial Community Analyses

#### DNA Extraction

Total genomic DNA was extracted by repeated bead beating (Mini-Bead-beater 8, BioSpec Inc., Bartlesville, United Kingdom) plus column purification method (Yu and Morrison, 2004). The yield and quality of extracted DNA was determined using a NanoDrop spectrophotometer (VWR International BVBA, Leuven, Belgium). Extracted DNA was used for bacterial 16S rRNA gene amplicon sequencing and quantitative real time PCR (qPCR).

For amplicon sequencing, extracted gDNA was submitted to Macrogen Sequencing Service (Macrogen, Seoul, South Korea) for library preparation and bacterial 16S rRNA gene amplicon sequencing (V3–V4 region, primers: 344F and 806R; Klindworth et al., 2013). Preparation of the amplicons barcoded library was based on the Illumina 16S metagenomic sequencing library preparation protocol^1^ and the sequencing was performed using Illumina MiSeq V3-technology (2 × 300 bp).

#### Bacterial 16S rRNA Gene Amplicon Sequencing: Pipeline and Data Treatment

The amplicon sequencing dataset was demultiplexed and barcodes were clipped off by the sequence provider. Reads were processed and analyzed using the Quantitative Insights into Microbial Ecology (QIIME1) bioinformatics pipeline version 1.9.1 (Caporaso et al., 2010b). Forward and reverse reads were merged using the fastq-join method (Aronesty, 2011), after which primer removal and quality filtering was performed using QIIME1. The subsequent analysis, picking Operational Taxonomic Units (OTU), assigning taxonomy, inferring phylogeny and creating OTU tables, were also performed by QIIME1. The sequences were clustered into OTU using the open-reference OTU picking workflow with a 97% similarity threshold using UCLUST, and chimeras were removed using UCHIME (Edgar, 2010). Representative OTU sequences were aligned to the Greengenes 97% core OTU set (v13_8; DeSantis et al., 2006), with a minimum percent identity of 97% using the PyNast algorithm (Caporaso et al., 2010a) with QIIME1 default parameters. Quality filtering resulted in an average 66 699 ± 11 067 reads per sample. Rarefaction analyses were performed using QIIME1, which indicated that the sequencing depth is enough to analyze the bacterial communities in all samples (data not shown). Both alpha diversity (Chao 1, PD whole tree, observed OTU, Shannon index, and dominance) and beta diversity (based on Bray–Curtis dissimilarity; Bray and Curtis, 1957) indices were determined using QIIME1. The principal coordinate (PCoA) plots were generated from Bray Curtis dissimilarity matrices, and the non-parametric permutational MANOVA-based statistical test ANOSIM and ADONIS were used to determine differences in overall microbial community between treatments. To analyze the differences in taxa abundance between treatments, a first screening was performed using Kruskal–Wallis test in QIIME1 (Caporaso et al., 2010b). Taxa that showed differences between treatment were further analyzed using a non-parametric factorial ANOVA approach using art function in ARTool R package version 0.10.7 (Wobbrock et al., 2011). Core successional microbes were generated using the QIIME software package with a script core_diversity_analyses.py. Sequence data have been deposited in the National Center for Biotechnology Information (NCBI) database under accession number PRJNA757729.

#### Quantification of Major Microbial Groups (Quantitative Real Time PCR)

The abundance of the 16S rRNA gene of total bacteria, *Ruminococcus flavefaciens*, *Ruminococcus albus*, *Fibrobacter succinogenes*, *Selenomonas ruminantium*, and *Synergistes jonesii*, 5.8S rRNA gene of anaerobic fungi (*Neocallimastigales*), and 18S rRNA gene of protozoa were quantified by qPCR. The primers used for the qPCR are given in Supplementary Table S4.

The qPCR reactions were assayed in a 12.5 μL reaction mixture containing 6.25 μL of Maxima^®^ SYBR Green/ROX qPCR Master Mix (2X) (ThermoFischer Scientific, Waltham, MA, United States), 1 μL of primer mixture containing 0.5 μM of each primer, DNA (20 ng) and molecular water. Amplification of each target group was carried out in a two-step cycling protocol (StepOne™ Real Time PCR System, Applied Biosystems, CA, United States) with the following program: initial denaturation at 95°C for 10 min, 35 cycles at 95°C for 15 s (denaturation), 60°C for 1 min (annealing/extension). The melting curve was built by measuring the fluorescence emissions with increased temperature from 60 to 95°C with ramps of 0.5°C every 15 s. Duplicate qPCR quantification was performed on 20 ng of extracted DNA. A plasmid containing a single copy of the targeted gene of each microorganism was used as qPCR standards for each target. The copy numbers in the standards were calculated based on the DNA concentrations determined by the NanoDrop. External standards were prepared and used in every qPCR run to enumerate the gene copies in the samples. The absolute quantity of each group of microorganisms was calculated using the respective standards and expressed as corresponding gene copies/mL of sample (Staroscik, 2004).

#### Colostrum, and Plasma Chitotriosidase Activity

Colostrum and plasma chitotriosidase (ChT) activity was measured as described by Arguello et al. (2008). Briefly, 1 μL of sample (either colostrum or plasma) was incubated with 100 μL of 22 mM artificial ChT substrate (4-methylumbelliferyl-D-*N*, *N′, N″* triacetylchitotriose, Sigma-Aldrich) in 0.5 M citrate phosphate buffer (pH 5.2) for 15 min at 37°C. The reaction was stopped by 5 mL of 0.5 M Na_2_CO_3_-NaHCO_3_ buffer (pH 10.7). Fluorescence was measured at 365 nm excitation and 450 nm emission (Fluorimeter Infinite 200, Tecan, Switzerland). The ChT activity was quantified as nanomoles of substrate hydrolyzed per milliliter (U/mL) (Moreno-Indias et al., 2012). Each sample was tested in triplicate and a valid result was considered when the standard deviation was less than 10% of the average.

#### Immunoglobulin M and Immunoglobulin G Concentrations in Saliva and Serum of Kids, and in Colostrum

Immunoglobulin M (IgM) and immunoglobulin G (IgG) were quantified as mg/mL of sample using commercial ELISA kits (Life Diagnostic Inc., West Chester, PA, United States). Pilot tests were performed with samples of each experimental condition to determine a suitable sample dilution fitting within the kit’s quantitation interval. For IgM quantitation, serum, saliva and colostrum were diluted at 1:25000, 1:500, and 1:25000, respectively. For IgG quantitation the dilutions were 1:150000, 1:2000, and 1:450000 for serum, saliva, and colostrum, respectively. Samples were individually tested in triplicate by measuring the optical density at 450 nm in microplate reader Infinite 200 (Tecan, Switzerland). A valid result was considered when the standard deviation was less than 10% of the average.

#### *In vivo* Assays to Assess the Feed Digestibility

The apparent dry matter (DM) and organic matter (OM) digestibility was assessed *in vivo* according to the recommendations of Azevedo et al. (2014). Briefly, three kids from each group were allocated in metabolic cages for 8 days at 20 weeks of age (3 days of adaptation). Animals were fed as described in the post-natal treatment section. All animals had free access to water. Feed refusals were collected just prior to the distribution of the next feeding. Feces were collected daily at 08:00 h from the fecal bags attached to the cage and weighed. Prior to the feces collection, kids were removed from the metabolic cages, which were cleaned completely. Urine and feces were separated and contact was avoided through a urine device collector (Lima et al., 2011). The refused feed and feces were collected and dried in an oven at 65°C for 72 h, pooled by animal/day, ground and sieved through a 1 mm screen, and stored until analysis.

Intake was calculated by difference between feed offered and feed refusal. Dry Matter Digestibility (DMD) and Digestible Organic Matter in DM (DOMD) were calculated based on Briceño-Poot et al. (2012).

### Statistical Analysis

A non-parametric factorial ANOVA approach using the art function in the ARTool R package version 0.10.7 (Wobbrock et al., 2011) in the R statistical computing environment (version 3.6.1) was used for the analysis of the data obtained. Prenatal treatment, postnatal treatment and their interaction were used as fixed effects, and mother’s identity (kids delivered by the same doe) was used as random factor. The BH procedure (Benjamini and Hochberg, 1995) was used for multiple comparisons and treatment effects were declared significant at *P* < 0.05 and a trend toward significance at 0.05 ≤ *P* < 0.10. In addition, Spearman Rank non-parametric correlation was performed to check the correlation between different taxa (at genus level) and the average daily gain [ADG], and FCE using SPSS 21.0 (SPSS, 2012).

## Results

### Age Is the Major Determinant of Variation in Rumen Bacterial Community Composition, Animal Performance and Immune Status

Diversity in the ruminal bacterial community structure of goat kids generally increased until 14 weeks of age, while dominance decreased (Supplementary Table S5). When all samples were visualized using a PCoA plot at bacterial OTU level, samples clustered based on the age of the kids (Supplementary Figure S1). Samples of 14-week and 20-week-old goat kids clustered together, and this cluster was away from the samples of the younger kids along PC1, which explained 35% of the variation. Bacterial OTU of 4- and 8-weeks-old kids clustered separately from each other along the PC2 axis, which only explained 8% of the variation. Particularly, samples from 8-weeks-old kids were very diverse, with some clustering separately from samples of 4-weeks-old kids while others were not separated.

**FIGURE 2:**
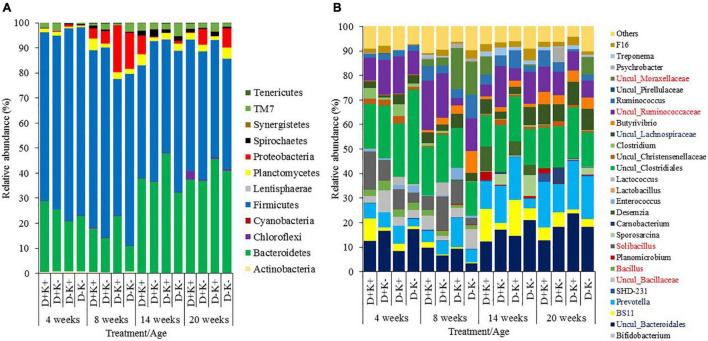
Relative abundance of rumen bacteria (%) in goat kids at phyla **(A)** and genus **(B)** level (relative abundance >1%) of goat kids at 4, 8, 14, and 20 weeks of age. Genera presented in the legend in blue or red font, indicate higher abundance at older or younger ages, respectively. Although the genera Solibacillus, Bacillus, Uncul_Bacillaceae were significantly higher in weeks 4 and 8 compared to weeks 14 and 20, this could not be visualized in weeks 14 and 20 due to too limited abundance.

In total, 12 bacterial phyla were identified across the four age groups, with Bacteroidetes and Firmicutes being the dominant phyla, irrespective of age (Figure 2). The relative abundance of the phylum Firmicutes was higher (*P* < 0.05) at the age of 4 and 8 weeks compared with 14 and 20 weeks, mainly due to the increased relative abundance of the genera *Solibacillus*, *Bacillus*, Uncul_Bacillaceae and Uncul_Ruminococcaceae at younger age, while Uncul_Lachnospiraceae were relatively more abundant in older kids. The relative abundance of the phylum Bacteroidetes remained similar at 4 and 8 weeks of age, but became more important (*P* < 0.05) at 14 and 20 weeks of age, mainly due to the increased relative abundance of the genus *Prevotella* and uncultured Bacteroidales.

The gene copy numbers of total bacteria, *Neocallimastigales* (fungi), protozoa, and specific bacteria were significantly influenced by age (Supplementary Table S6). Virtually, no protozoa were present in the rumen fluid of kids at 4 and 8 weeks of age, irrespective of the experimental treatment. The 16S rRNA gene copy number of total bacteria increased (*P* < 0.05) after weaning (14 and 20 weeks) compared with 4 and 8 weeks. The copy numbers of *Neocallimastigales* remained similar at 4 and 8 weeks, decreased (*P* < 0.05) at 14 weeks, but increased to the level of 4 weeks at the age of 20 weeks. Additionally, *R. flavefaciens* and *S. ruminantium* were higher (*P* < 0.05) after weaning compared with preweaning. The 16S rRNA gene copy numbers of *S. ruminantium* and *F. succinogenes* were highest (*P* < 0.05) at 14 weeks. The abundance of 16S rRNA genes of *R. albus* was higher (*P* < 0.05) after weaning compared with 4 weeks. The copy numbers of *S. jonesii* increased (*P* < 0.05) till 14 weeks, but decreased to the level of 8 weeks at the age of 20 weeks.

Additionally, some of the studied animal performance parameters (BW, DMI, and FCE) increased with age as expected (*P* < 0.05, Supplementary Table S7). The immune status of kids (Total WBC, eosinophils, monocytes, ChT activity, and the concentration of IgG and IgM in serum and saliva) was also influenced by age (*P* < 0.05), except for the lymphocyte and neutrophil proportions.

### Effects of Early Life Nutritional Intervention on Bacterial Community Composition Were More Evident After Weaning

The postnatal treatment continued until weaning. After weaning, all kids received *L. leucocephala* forage meal (30% of the requirements of CP) mixed with a commercial concentrate twice a day. The effects of prenatal and postnatal treatments on bacterial community composition were more evident after weaning (14 and 20 weeks), once the postnatal treatment was ceased.

Generally, prenatal treatment did not influence any of the alpha diversity indices measured (Table 1). However, at the age of 8 weeks, dominance tended to be lower in D+ kids as compared with D– kids (*P* = 0.08). A postnatal effect was observed in alpha diversity indices only at 20 weeks, 12 weeks after the treatment ceased: the Shannon index was higher and the dominance was lower in K+ kids as compared with K– kids (*P* < 0.05). Furthermore, interaction effects were observed on alpha diversity indices at the age of 4 weeks: diversity (PD_Whole tree) and richness (Chao1 index) were (*P* < 0.05) or tended to be (*P* = 0.09) higher in D+K+ and D–K– kids compared with D+K– kids, while dominance was higher in D+K+ kids compared with D–K+ kids.

**TABLE 1:** Mean (standard deviation) of α-diversity indices (Chao1, PD_Whole_tree, Observed OTU, Shannon index and dominance) characterizing the rumen bacterial community structure of goat kids at 4, 8, 14, and 20 weeks of age.

Alpha diversity indices	Treatments				*P*-value		
	D+K+	D+K–	D–K+	D–K–	Prenatal	Postnatal	Interaction
	**4 weeks**						

Chao1	716 (156.2)	629 (228.6)	671 (80.2)	738 (49.6)	0.555	0.501	0.094
PD_Whole_tree	67.5^a^ (14.22)	58.1^b^ (18.86)	63.6^ab^ (7.80)	68.0^a^ (3.25)	0.455	0.466	0.042
Observed OTU	631 (158.3)	566 (208.1)	613 (78.9)	662 (27.2)	0.364	0.671	0.149
Shannon index	5.59 (0.628)	5.89 (0.917)	6.30 (0.308)	5.80 (0.261)	0.356	0.830	0.290
Dominance	0.10^a^ (0.049)	0.06^ab^ (0.032)	0.04^b^ (0.011)	0.08^ab^ (0.014)	0.507	0.918	0.046

	**8 weeks**						

Chao1	820 (151.9)	829 (161.7)	782 (82.3)	786 (33.3)	0.409	0.853	0.248
PD_Whole_tree	76.2 (11.86)	75.1 (13.31)	70.1 (7.12)	70.8 (1.67)	0.278	0.178	0.801
Observed OTU	746 (130.2)	734 (170.7)	673 (93.3)	701 (27.6)	0.372	0.430	0.747
Shannon index	6.29 (0.385)	6.01 (0.882)	5.32 (0.452)	5.81 (0.654)	0.148	0.851	0.109
Dominance	0.04 (0.012)	0.06 (0.057)	0.09 (0.024)	0.08 (0.059)	0.084	0.361	0.114

	**14 weeks**						

Chao1	1016 (69.7)	967 (68.2)	999 (56.8)	984 (79.2)	0.990	0.378	0.631
PD_Whole_tree	90.7 (6.48)	88.6 (5.13)	91.1 (5.15)	89.6 (6.54)	0.699	0.645	0.942
Observed OTU	908 (95.3)	876 (62.3)	908 (72.0)	890 (66.3)	0.751	0.521	0.782
Shannon index	6.17 (0.839)	6.63 (0.155)	6.47 (0.398)	6.43 (0.817)	0.798	0.407	0.619
Dominance	0.07 (0.045)	0.04 (0.003)	0.05 (0.009)	0.06 (0.051)	0.772	0.414	0.888

	**20 weeks**						

Chao1	991 (39.0)	970 (37.4)	1035 (124.0)	969 (46.0)	0.669	0.318	0.745
PD_Whole_tree	89.7 (2.68)	89.9 (3.42)	94.3 (8.28)	89.5 (3.62)	0.446	0.440	0.475
Observed OTU	895 (35.6)	883 (42.5)	942 (101.0)	889 (41.5)	0.497	0.398	0.635
Shannon index	6.83 (0.161)	6.17 (0.553)	6.92 (0.189)	6.58 (0.437)	0.209	0.001	0.179
Dominance	0.03 (0.006)	0.07 (0.035)	0.03 (0.005)	0.04 (0.023)	0.200	0.009	0.186

*Different letters in the same row (^a, b^) indicate significant differences among treatments (P < 0.05) according to the BH procedure (Benjamini and Hochberg, 1995).*

**FIGURE 3:**
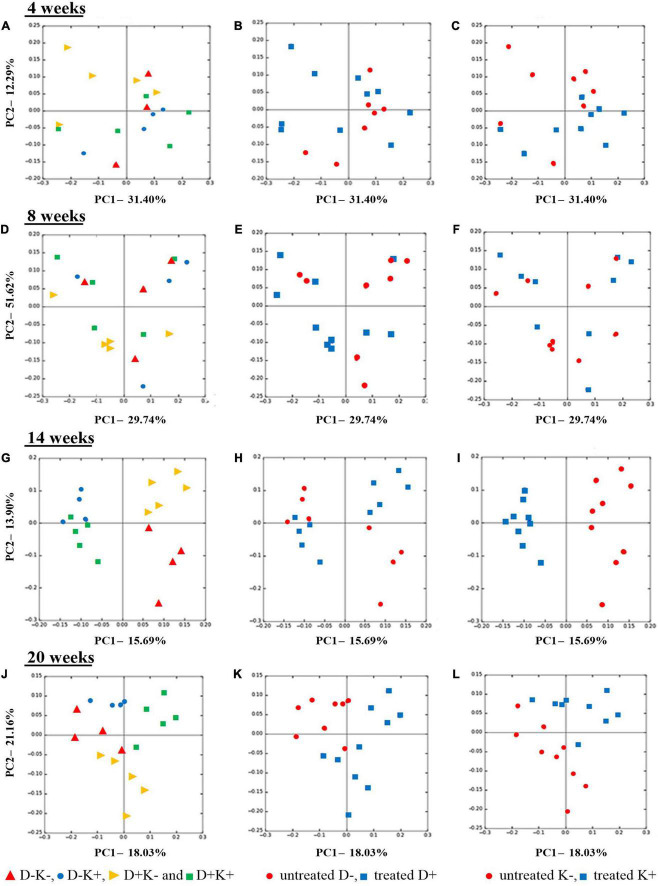
Principal coordinate analysis (PCoA), based on Bray-Curtis dissimilarity indices at OTU level, of the rumen bacterial community structure of goat kids pre weaning (4 weeks), at weaning (8 weeks) and post weaning (14 and 20 weeks). Kids were treated prenatally through supplementation of *L. leucocephala* forage meal in their mothers’ diet during the last 7 weeks of pregnancy (D+), postnatally until weaning with yeast (K+), or not (D− and K−). Left Figures **(A,D,G,J)** present all four treatments separately while middle **(B,E,H,K)** and right **(C,F,I,L)** figures visualize prenatal and postnatal treatments, respectively.

There were no prenatal, postnatal nor interaction effects on the bacterial community structure at 4 and 8 weeks (Figures 3A–F), which was confirmed by the ANOSIM and ADONIS analysis (*P* > 0.05). However, at 4 weeks of age, the K+ kids showed lower PC2 values (close to 0 or negative), except for one observation. In contrast, K– kids showed positive PC2 values, except for 2 observations. Inter-animal variation within each treatment seemed to be higher in younger animals as compared with animals at post-weaning ages (Figures 3G–L). Indeed, treatment effects were more evident at 14 weeks (Figures 3G–I) and 20 weeks (Figures 3J–L), which was confirmed by ANOSIM and ADONIS analysis (*P* < 0.05), except for the prenatal effect at 14 weeks (*P* = 0.116). At 14 weeks of age, K+ kids clustered away from K– kids along the PC1 axis, which explained 16% of the variation (Figure 3I). Furthermore, K+ kids showed lower individual variability as compared with the K– group at this age (Figure 3I). A clear clustering was also observed between D+K– and D–K– kids along the PC2 axis (13.9% of variation explained) in which D–K– kids were more diverse compared with D+K– kids (Figure 3G). At 20 weeks, clustering according to the prenatal treatment (D+ *vs.* D–) was obvious along the PC1 axis (Figure 3K), in which the majority of the D+ kids showed a positive PC1 value and high individual variation, whereas all D– kids showed lower PC1 values (negative or closer to 0) and less individual variation. Notably, at the age of 20 weeks, D+K+ kids clustered away from the other treatment groups along the PC1 axis, which explained 18% of the variation (Figure 3J). The other three groups clustered separately from each other along the PC2 axis, which explained 12% of the variation.

Differences in the relative abundance of bacteria were analyzed at phylum and genus levels (Figure 2). Statistics are presented separately by age group in Supplementary Table S8, where only taxa representing more than 1% of the total bacterial community in at least one treatment group are shown. No effects of treatments were observed at phylum level and only few genera showed differences at 4 and 8 weeks of age. At 4 weeks, the relative abundance of Uncul_Lachnospiraceae was higher (*P* < 0.05), and the relative abundance of Uncul_Bacteroidales tended to be lower (*P* = 0.07) in K+ kids compared with K– kids. Additionally, the relative abundance of Uncul_Lachnospiraceae tended to be higher (*P* = 0.07) in D– kids compared with the D+ kids. At 8 weeks, the relative abundance of the genus *Clostridium* was higher in D+ kids (*P* < 0.05) compared with D– kids. The relative abundance of *Butyrivibrio* was lower (*P* = 0.05), while BS11 tended to be higher (*P* = 0.07) in K+ kids compared with K– kids.

After weaning, treatment differences were observed at phylum and genus levels, although all animals were given the same diet (Figure 2 and Supplementary Table S8). No prenatal effects were observed at 14 weeks. Firmicutes were affected by the postnatal treatment and tended to be lower in K+ kids compared with K– kids (*P* = 0.07) at 14 weeks of age. At genus level, K+ kids showed higher (*P* < 0.05) relative abundance of BS11 and lower abundance of Uncul_Ruminococcaceae as compared with K– kids. Additionally, the abundance of the genus *Desemzia* tended to be higher (*P* = 0.07) and of Uncul_Christensenellaceae tended to be lower (*P* = 0.10) in K+ kids compared with K- kids. Moreover, some interactions were observed: *Prevotella* and Uncul_Lachnospiraceae showed the highest and lowest abundance, respectively, in untreated kids (D–K–), while single treated kids (D+K– and D–K+) showed the lowest and highest abundances. Inversely, D+K+ kids showed the lowest abundance of Uncul_Clostridiales, while single treated kids showed the highest abundances.

At 20 weeks, the relative abundance of the phylum Chloroflexi was higher (*P* > 0.05) in D+ kids and tended to be higher (*P* = 0.06) in K+ kids compared with D– and K– kids, respectively, particularly due to the higher (*P*_*interaction*_ < 0.05) relative abundance of this phylum in D+K+ kids compared with D+K– kids. The relative abundance of the phylum TM7 was higher in K+ kids (*P* < 0.05) compared with K– kids, particularly in prenatally untreated kids (*P_*interaction*_* = 0.06). In the current microbial dataset, only one genus has been identified within the phylum TM7 and Chloroflexi (i.e., F16 and SHD-231, respectively). As such these genera determined the effect of their respective phyla. The relative abundance of the phylum Proteobacteria tended to be higher (*P* = 0.08) in K– kids compared to K+ kids. At the age of 20 weeks, the genus *Ruminococcus* showed a higher (*P* < 0.05) relative abundance in D+ kids compared with D– kids and also tended to be higher (*P* = 0.05) in D+K+ kids compared with D–K+ and D–K– kids. The genera *Prevotella* tended to be higher (*P* = 0.07) and Uncul_Christensenellaceae tended to be lower (*P* = 0.05) in K+ compared with K– kids at the age of 20 weeks. The latter was in line with the observation at 14 weeks, while other postnatal differences at 14 and 20 weeks varied.

The qPCR data suggested neither the bacterial nor fungal (*Neocallimastigales*) numbers were affected by the treatments at younger ages (Table 2). Protozoa were observed only after weaning and were higher in K+ kids compared with K– kids at 14 and 20 weeks. Protozoa numbers were also higher in D+K+ kids compared with K– kids (D+K– and D–K–) at 14 weeks. 16S rRNA gene copies of *F. succinogenes* and *R. albus* tended to be higher (*P* = 0.10 and *P* = 0.09, respectively) in K+ kids compared with K– kids at 4 weeks of age. At the age of 8 weeks, 16S rRNA gene copies of *R. albus* tended to be higher in D+ kids compared with D– kids (*P* = 0.10). From 14 weeks of age onward, some differences were observed in the quantitative abundance of *S. jonesii*, but the prenatal *L. leucocephala* supplementation to mother goats did not enhance the abundance of this genus. Also at 20 weeks of age, the abundance of *S. ruminantium* was higher (*P* < 0.05) in K+ kids compared with K– kids and tended to be higher in D+ kids (*P* = 0.09) compared with D– kids. Additionally, the quantity of *S. ruminantium* was highest in D+K+ kids.

**TABLE 2:** Mean (standard deviation) of absolute abundance of target rumen microbes determined by real-time PCR (log_10_/mL rumen fluid) of goat kids at 4, 8, 14, and 20 weeks of age.

Index	Treatments				*P*-value		
	D+K+	D+K–	D–K+	D–K–	Prenatal	Postnatal	Interaction
	**4 weeks**						

Total Bacteria	12.6 (0.17)	12.5 (0.18)	12.4 (0.31)	12.3 (0.15)	0.247	0.583	0.798
Fungi^1^	9.8 (0.19)	9.8 (0.26)	9.7 (0.39)	9.4 (0.21)	0.128	0.408	0.380
Protozoa	Nd	nd	nd	nd	–	–	–
*F. succinogenes*	10.2 (0.81)	9.22 (0.84)	10.3 (0.43)	10.3 (0.41)	0.250	0.096	0.218
*R. flavefasciens*	8.7 (0.50)	8.1 (1.77)	9.1 (0.79)	9.2 (0.64)	0.306	0.732	0.701
*R. albus*	9.7 (1.87)	7.9 (1.52)	9.1 (1.73)	7.9 (0.75)	0.872	0.088	0.769
*S. ruminantium*	11.7 (0.45)	11.5 (0.32)	11.6 (0.33)	11.5 (0.42)	0.785	0.355	0.788
*S. jonesii*	8.9 (0.53)	8.0 (1.05)	8.7 (0.38)	8.5 (0.63)	0.806	0.274	0.400

	**8 weeks**						

Total Bacteria	12.6 (0.19)	12.6 (0.20)	12.6 (0.19)	12.5 (0.28)	0.671	0.659	0.283
Fungi	9.8 (0.19)	10.3 (1.07)	10.2 (1.15)	9.7 (0.14)	0.727	0.939	0.427
Protozoa	nd	nd	nd	nd	–	–	–
*F. succinogenes*	10.5 (0.59)	10.9 (1.03)	10.9 (0.80)	10.5 (0.54)	0.671	0.659	0.283
*R. flavefasciens*	8.5 (0.60)	8.2 (0.71)	9.2 (0.73)	9.0 (0.77)	0.727	0.939	0.427
*R. albus*	9.8 (0.55)	10.3 (1.06)	9.0 (1.30)	8.5 (1.29)	0.105	0.835	0.190
*S. ruminantium*	11.4 (0.23)	11.5 (0.39)	11.4 (0.23)	11.5 (0.44)	0.969	0.652	0.912
*S. jonesii*	8.7 (0.82)	8.6 (0.93)	9.1 (0.39)	8.9 (0.50)	0.450	0.862	0.972

	**14 weeks**						

Total Bacteria	12.9 (0.25)	13.1 (0.37)	13.2 (0.22)	12.9 (0.33)	0.700	0.785	0.205
Fungi	8.0 (0.51)	8.1 (0.50)	8.0 (0.52)	7.6 (0.34)	0.510	0.217	0.177
Protozoa	11.1^a^ (0.42)	9.74^c^ (0.31)	10.8^ab^ (0.07)	10.5^b^ (0.19)	0.159	<0.001	0.001
*F. succinogenes*	11.0 (0.43)	11.2 (0.51)	11.4 (0.77)	10.9 (0.58)	0.701	0.817	0.351
*R. flavefasciens*	9.0 (0.46)	10.1 (0.73)	9.8 (0.91)	9.3 (1.12)	0.818	0.310	0.083
*R. albus*	10.1 (0.30)	10.8 (0.69)	10.5 (0.63)	10.0 (0.71)	0.535	0.536	0.149
*S. ruminantium*	12.0 (0.29)	12.4 (0.36)	12.4 (0.26)	12.1 (0.40)	0.979	0.505	0.080
*S. jonesii*	9.4^b^ (0.26)	9.7^a^ (0.15)	9.7^a^ (0.15)	9.5^ab^ (0.29)	0.911	0.404	0.033

	**20 weeks**						

Total Bacteria	13.0 (0.22)	12.9 (0.11)	12.9 (0.13)	12.9 (0.12)	0.412	0.812	0.401
Fungi	9.8 (0.38)	9.3 (0.35)	9.5 (0.28)	9.4 (0.18)	0.537	0.117	0.327
Protozoa	11.3 (0.38)	10.5 (0.51)	11.1 (0.50)	10.7 (0.26)	0.803	0.008	0.510
*F. succinogenes*	10.6 (0.34)	10.2 (0.20)	10.7 (0.35)	10.6 (0.33)	0.121	0.248	0.296
*R. flavefasciens*	9.7 (0.68)	9.5 (0.60)	9.6 (1.15)	9.2 (0.49)	0.757	0.265	0.657
*R. albus*	10.1 (0.54)	9.8 (0.13)	9.9 (0.22)	9.9 (0.73)	0.881	0.455	0.505
*S. ruminantium*	12.2^a^ (0.18)	11.7^b^ (0.16)	11.7^b^ (0.21)	11.8^b^ (0.17)	0.089	0.016	0.005
*S. jonesii*	8.6^bc^ (0.07)	8.5^c^ (0.10)	8.7^a^ (0.09)	8.6^b^ (0.19)	<0.001	<0.001	<0.001

*Values with different superscripts in same row (^a, b, c^) differ significantly (P < 0.05) among treatments according to the BH procedure (Benjamini and Hochberg, 1995).*

*^1^Fungi was determined based on the order Neocallimastigales.*

### Core Successional Microbiome

The core microbiome (present in 80% of all animals) was characterized at the age of 4, 8, 14, and 20 weeks irrespective of the treatment (Supplementary Figure S2). In total, eight core phyla were identified across the four age groups. Similar to the overall microbiome, core Firmicutes and Bacteroidetes were the dominant phyla irrespective of age. Other predominant core bacteria (relative abundance > 1% in at least one of the age groups) belonged to the phyla Planctomycetes and TM7. Contrary to the overall observations, the relative abundance of core Bacteroidetes decreased with age (*P* < 0.01) mainly as a result of the decreasing relative abundance of core OTU within the genera Uncul_Bacteroidales and *Prevotella*, except at 14 weeks of age where an increase was observed of the relative abundance of the core BS11 genus (phylum Bacteroidetes). In contrast, the relative abundance of core Firmicutes increased with age (*P* < 0.01), except at 14 weeks of age mainly due to the decreased relative abundance of the core OTU within the genus Uncul_Clostridiales.

Only 49 OTU, observed at the age of 4 weeks, persisted until 20 weeks. These core OTU belonged to three phyla: Actinobacteria, Bacteroidetes, and Firmicutes (Table 3). The relative distribution of the core-persistent microbiome mainly depended on the age (Supplementary Table S9), but some bacterial phyla and genera showed prenatal and/or postnatal treatment effects. In contrast to the overall bacteriome, at phylum level, there was a lower relative abundance (*P* < 0.05) of core Bacteroidetes (solely represented by OTU of the genus Uncul_Bacteroidales) in K+ kids as compared with K– kids at 4 weeks of age. Core Firmicutes showed an interaction effect at the age of 4 weeks and were relatively least abundant in D+K+ kids (*P* < 0.05). This was mainly due to a tendency for lower relative abundance of the core OTU in the family Uncul_Clostridiales (*P* = 0.08). Additionally, the relative abundance of the core Uncul_Ruminococcaceae at 4 weeks of age was lowest in D–K– (*P* < 0.05). The relative abundance of core-bacteria belonging to the genera *Ruminococcus, Oscillospira*, and Uncul_Lachnospiraceae, Ruminococcaceae, Mogibacteriaceae, Christenellaceae, Clostridiaceae, showed some treatment differences at 8, 14, or 20 weeks of age (Table 3). The relative abundance of the core OTU belonging to the genera Uncul_Lachnospiraceae and Uncul_Ruminococcaceae was increased by the postnatal treatment at week 20, 12 weeks after the treatment was ceased. Core OTU of the genus Uncul_Coriobacteriaceae (phylum Actinobacteria) colonized the rumen at 4 weeks but their abundance decreased with age (Supplementary Table S9) while no treatment effect was observed.

**TABLE 3:** Mean (standard deviation) of the relative abundance (%) of the core-persistent microbiome (phyla [in **bold**] and genus level) which were observed in 80% of the goat kids irrespective of treatment at 4 weeks of age and that persisted through 8 and 14 weeks of age till 20 weeks of age.

Taxa	Treatments				*P*-value		
	D+K+	D+K–	D–K+	D–K–	Prenatal	Postnatal	Interaction
	**4 weeks**						

**Actinobacteria** (Uncul_Coriobacteriaceae*)	0.1 (0.11)	0.1 (0.09)	0.2 (0.18)	0.5 (0.56)	0.267	0.394	0.216
**Bacteroidetes** (Uncul_Bacteroidales*)	1.7 (1.34)	11.9 (8.18)	4.1 (4.40)	7.8 (13.73)	0.614	0.030	0.204
**Firmicutes**	9.5^b^ (2.80)	14.7^a^ (2.93)	18.2^a^ (6.06)	11.6^ab^ (7.64)	0.564	0.589	0.032
Uncul_Clostridiales	4.9 (2.12)	9.9 (3.47)	8.1 (3.69)	6.4 (4.69)	0.938	0.265	0.083
Uncul_Mogibacteriaceae	0.1 (0.04)	0.4 (0.03)	0.1 (0.04)	0.0 (0.01)	0.900	0.089	0.078
Uncul_Christensenellaceae	0.8 (0.70)	1.5 (1.15)	1.3 (1.86)	0.7 (0.91)	0.433	0.605	0.299
Uncul_Clostridiaceae	0.1 (0.09)	0.1 (0.14)	0.1 (0.06)	0.0 (0.03)	0.412	0.167	0.624
Uncul_Lachnospiraceae	1.4 (1.16)	0.2 (0.40)	1.9 (1.59)	1.7 (1.68)	0.218	0.168	0.137
Uncul_Ruminococcaceae	2.1^b^ (1.56)	2.5^b^ (0.90)	6.2^a^ (5.09)	1.5^b^ (1.84)	0.084	0.054	0.043
Oscillospira	0.0 (0.03)	0.1 (0.09)	0.0 (0.01)	0.0 (0.04)	0.688	0.553	0.724
Ruminococcus	0.0 (0.05)	0.4 (0.80)	0.4 (0.39)	1.2 (0.73)	0.060	0.018	0.307
Other_Ruminococcaceae	0.0 (0.03)	0.0 (0.01)	0.0 (0.01)	0.0 (0.00)	0.718	0.241	0.786

	**8 weeks**						

**Actinobacteria** (Uncul_Coriobacteriaceae*)	0.1 (0.13)	0.0 (0.01)	0.1 (0.10)	0.1 (0.12)	0.578	0.508	0.445
**Bacteroidetes** (Uncul_Bacteroidales*)	1.4 (1.58)	1.0 (1.52)	1.5 (1.84)	0.1 (0.06)	0.841	0.274	0.644
**Firmicutes**	11.7 (3.47)	16.8 (16.69)	11.9 (8.73)	10.7 (3.30)	0.510	0.418	0.801
Uncul_Clostridiales	5.3 (1.73)	6.5 (5.22)	7.4 (9.70)	4.6 (1.70)	0.628	0.859	0.567
Uncul_Mogibacteriaceae	0.1 (0.07)	0.1 (0.07)	0.1 (0.05)	0.2 (0.24)	0.577	0.403	0.192
Uncul_Christensenellaceae	0.2 (0.11)	0.3 (0.28)	0.1 (0.05)	0.1 (0.03)	0.017	0.477	0.561
Uncul_Clostridiaceae	0.3^a^ (0.20)	0.1^b^ (0.11)	0.2^ab^ (0.31)	0.2^ab^ (0.17)	0.849	0.440	0.012
Uncul_Lachnospiraceae	1.1 (0.96)	0.6 (0.35)	2.4 (1.99)	1.3 (0.56)	0.064	0.213	0.447
Uncul_Ruminococcaceae	1.3 (1.19)	6.7 (12.21)	0.6 (0.37)	0.8 (0.82)	0.402	0.502	0.473
Oscillospira	0.0 (0.02)	0.0 (0.03)	0.0 (0.01)	0.0 (0.02)	0.199	0.967	0.543
Ruminococcus	3.2 (5.03)	2.1 (2.54)	1.2 (2.01)	3.5 (4.80)	0.872	0.936	0.838
Other_Ruminococcaceae	0.0 (0.01)	0.3 (0.61)	0.0 (0.01)	0.0 (0.04)	0.424	0.410	0.407

	**14 weeks**						

**Actinobacteria** (Uncul_Coriobacteriaceae*)	0.0 (0.01)	0.0 (0.01)	0.0 (0.01)	0.1 (0.12)	0.400	0.093	0.150
**Bacteroidetes** (Uncul_Bacteroidales*)	0.6 (0.55)	0.4 (0.27)	0.3 (0.24)	0.2 (0.13)	0.261	0.861	0.845
**Firmicutes**	10.8 (4.86)	10.7 (4.72)	9.24 (1.475)	11.2 (4.01)	0.855	0.864	0.787
Uncul_Clostridiales	6.4 (1.88)	7.1 (3.90)	7.3 (1.51)	5.7 (1.49)	0.827	0.224	0.223
Uncul_Mogibacteriaceae	0.1 (0.05)	0.1 (0.04)	0.0 (0.02)	0.0 (0.01)	0.986	0.938	0.819
Uncul_Christensenellaceae	0.2 (0.13)	0.2 (0.13)	0.2 (0.15)	0.2 (0.11)	0.707	0.402	0.642
Uncul_Clostridiaceae	0.1 (0.10)	0.2 (0.16)	0.0 (0.02)	0.2 (0.18)	0.371	0.045	0.609
Uncul_Lachnospiraceae	2.8^a^ (2.73)	0.6^b^ (0.191)	0.6^ab^ (0.30)	3.0^a^ (3.05)	0.727	0.536	0.006
Uncul_Ruminococcaceae	0.9 (0.29)	2.7 (1.50)	0.9 (0.17)	2.0 (1.59)	0.210	0.001	0.195
Oscillospira	0.0 (0.02)	0.1 (0.02)	0.0 (0.01)	0.0 (0.02)	0.099	0.010	0.726
Ruminococcus	0.4 (0.36)	0.8 (1.09)	0.2 (0.19)	0.8 (0.47)	0.632	0.228	0.669
Other_Ruminococcaceae	0.0 (0.03)	0.1 (0.03)	0.0 (0.06)	0.1 (0.06)	0.584	0.086	>0.999

	**20 weeks**						

**Actinobacteria** (Uncul_Coriobacteriaceae*)	0.0 (0.01)	0.0 (0.01)	0.0 (0.01)	0.0 (0.03)	0.936	0.643	0.544
**Bacteroidetes** (Uncul_Bacteroidales*)	0.6^a^ (0.19)	0.3^b^ (0.18)	0.4^b^ (0.19)	0.5^ab^ (0.25)	0.878	0.161	0.045
**Firmicutes**	14.0 (4.93)	12.9 (4.38)	17.2 (7.44)	10.7 (2.97)	>0.999	0.279	0.471
Uncul_Clostridiales	7.9 (4.99)	8.7 (3.79)	10.2 (7.82)	7.1 (2.75)	0.877	0.871	0.434
Uncul_Mogibacteriaceae	0.0 (0.03)	0.1 (0.02)	0.1 (0.02)	0.1 (0.09)	0.082	0.008	0.377
Uncul_Christensenellaceae	0.5 (0.41)	0.6 (0.32)	0.6 (0.28)	0.4 (0.12)	0.914	0.956	0.213
Uncul_Clostridiaceae	0.1 (0.05)	0.1 (0.05)	0.1 (0.06)	0.1 (0.03)	0.728	0.578	0.927
Uncul_Lachnospiraceae	2.1 (1.46)	1.4 (0.92)	3.5 (0.96)	1.7 (0.52)	0.138	0.030	0.462
Uncul_Ruminococcaceae	2.8 (0.70)	1.7 (0.75)	2.6 (1.12)	1.1 (0.49)	0.533	0.015	0.342
Oscillospira	0.0 (0.03)	0.0 (0.02)	0.0 (0.02)	0.0 (0.04)	0.878	0.521	0.426
Ruminococcus	0.16 (0.176)	0.17 (0.168)	0.03 (0.027)	0.02 (0.035)	0.020	0.895	0.772
Other_Ruminococcaceae	0.17 (0.109)	0.07 (0.037)	0.17 (0.063)	0.12 (0.106)	0.588	0.106	0.858

*Values with different superscripts in same row (^a, b^) differ significantly (P < 0.05) among treatments according to the BH procedure (Benjamini and Hochberg, 1995).*

*The phyla of Actinobacteria and Bacteroidetes, only include one core genus, i.e., Uncul_Coriobacteriaceae and Uncul_Bacteroidales, respectively.

### Animal Performance and Immune Status

Animal performance data over the complete trial are presented in Table 4, while detailed data at intermediate ages are shown in Supplementary Table S10. Birth weight of kids was not affected by feeding *L. leucocephala* to mother goats in the last seven weeks of gestation. Both prenatal and postnatal treatments influenced the body weight in this trial. Throughout the whole experiment, D+ kids were heavier (*P* < 0.05) or tended to be heavier (at 4 weeks) compared with D– kids. Body weight was higher in K+ kids at 20 weeks (*P* < 0.05) and tended to be higher at 4 (*P* = 0.06) and 14 weeks (*P* = 0.07) compared with K– kids. Growth was higher in D+K+ kids (*P* = 0.05) compared with the other treatment groups in the first 4 weeks of life. Moreover, ADG was higher in K+ as compared with K– kids (*P* < 0.05) in the first 4 weeks and over the 20 weeks of life, and tended to be higher over the 14 weeks of life (*P* = 0.10) in K+ kids compared with K– kids. The DMI tended to be lower in D–K+ group of kids as compared with the other groups at this age. After weaning, the DMI were higher (*P* < 0.05) and tended to be higher (*P* = 0.07) in D+ compared with D– kids at 20 and 14 weeks, respectively. The FCE was higher (*P* < 0.05) in K– kids compared with K+ kids throughout the experimental period, except at the age of 8 weeks.

**TABLE 4:** Mean (standard deviation) of body weight (BW) of goat kids at 20 weeks of age and average daily gain (ADG), dry matter intake (DMI) and feed conversion efficiency (FCE) over the first 20 weeks of life (all kids) as well as mean (standard deviation) of DM, OM and digestible OM intake and apparent digestibility of DM and OM obtained during the digestibility trial of 5 days at 20 weeks of age (*n* = 3 per treatment).

Items	Treatments				*P*-value		
	D+K+	D+K–	D–K+	D–K–	Prenatal	Postnatal	Interaction
**Performance parameters (all animals)**							
BW (kg) (20 weeks)	16.2 (1.00)	14.1 (1.16)	14.5 (1.00)	13.2 (0.48)	0.024	0.006	0.456
ADG (g/d) (birth-20 weeks)	92.4 (5.89)	78.5 (6.95)	81.6 (6.99)	75.6 (4.31)	0.046	0.004	0.445
DMI (g/d) (birth-20 weeks)	418 (25.8)	393 (36.7)	371 (26.4)	368 (22.3)	0.050	0.135	0.761
FCE (kg/kg) (birth-20 weeks)	4.8 (0.10)	5.3 (0.13)	4.8 (0.12)	5.2 (0.14)	0.914	<0.001	0.945
**Digestibility trial (*n* = 3 per treatment)**							
**Intake (g/d)**							
DM	1036 (2.7)	964 (96.6)	886 (70.2)	863 (8.9)	0.003	0.195	0.500
OM	951 (72.4)	885 (102.2)	814 (81.6)	792 (39.5)	0.112	0.591	>0.999
Digestible OM	655 (19.6)	533 (88.1)	515 (45.0)	460 (21.5)	0.009	0.007	0.409
**Apparent digestibility (g/kg)**							
DM	709 (17.9)	629 (38.4)	657 (4.8)	608 (19.7)	0.077	0.001	0.262
OM	633 (17.3)	554 (37.7)	581 (4.7)	533 (19.4)	0.077	0.001	0.262

*DM, dry matter; OM, organic matter.*

The total WBC and their components, mainly monocytes and eosinophils, were also influenced by prenatal and postnatal treatments. Notably, the total WBC count was higher in 8- and 20-weeks old K+ kids, as well as in D+ kids at 20 weeks as compared with K– kids and D– kids, respectively (*P* < 0.05). The concentration of serum IgG (sIgG) and IgM [in serum (sIgM) and saliva (mIgM)] and chitotriosidase activity were influenced by prenatal and postnatal treatments (Supplementary Table S10). In particular, the concentration of sIgG was higher in D– and K+ kids at 8 and 20 weeks compared with D+ and K– kids. At 4 weeks, D+K+ and D–K– kids had higher concentrations of sIgG compared with the other two groups (Supplementary Table S10). In general, an opposite effect was observed in the concentration of mIgG (salivary IgG). The K+ kids showed higher concentrations of sIgM at 4 and 8 weeks and lower concentrations of sIgM at 20 weeks compared with K– kids. The concentrations of sIgM were higher in D+ kids at 4 weeks mainly due to the higher concentration of sIgM in D+K+ kids compared with the other groups. The concentrations of mIgM were higher in K+ and D+ kids at 8 weeks compared with K– and D– kids. An opposite effect was observed at 20 weeks: the concentration of mIgM was higher in K– and D– kids compared with K+ and D+ kids. There were also some interaction effects in the concentrations of sIgM and mIgM. The ChT activity was higher in D+K+ and D–K– kids compared with the other two groups at 4 weeks. At 8 weeks of age, the ChT activity was lower in D–K+ kids compared with D+K+ and D–K– kids (*P* < 0.05), while the ChT activity was higher in D+K+ compared with D+K– kids (*P* < 0.05) at 20 weeks. The ChT activity was higher in D– kids at 4 weeks and K+ kids at 20 weeks compared with D+ and K– kids, respectively.

Correlation analysis between the relative abundances of core bacterial genera (present in 80% of the animals) with ADG and FCE are given in Supplementary Table S11. At the age of 4 and 8 weeks, there were some positive and negative correlations between the relative abundance of some of the minor core genera with ADG and FCE. Among the major genera, some correlations were observed at 14 and 20 weeks of age, but none of the correlations were consistent at different time points: BS11 negatively correlated with FCE and Uncul_Ruminococcaceae correlated positively with FCE at 14 weeks of age. At 20 weeks, Uncul_Clostridiales and Uncul_Ruminococcaceae correlated positively with ADG, while Uncul_Bacteroidales, *Coprococcus* and *Pseudobutyrivibrio* correlated negatively. Additionally, Uncul_Clostridiales correlated negatively while Uncul_Bacteroidales correlated positively with FCE.

### *In vivo* Digestibility Trial

The *in vivo* digestibility assay was performed at the age of 20 weeks to assess the effects of the early-life intervention on the digestibility of diets with 30% of the dietary protein provided by *L. leucocephala* forage meal (Table 4). The DM intake and the digestible OM intake were higher in D+ kids compared with D– kids (*P* < 0.01), while the apparent digestibility of DM and the apparent digestibility of OM tended to be higher in D+ kids (*P* = 0.08). Postnatally treated kids (K+) showed higher digestible OM intake, and higher apparent digestibility of DM and OM compared with K– kids (*P* < 0.01). Furthermore, no interaction effects on the intake nor digestibility of DM or OM were observed.

## Discussion

Because of the resistance of the indigenous rumen microbiome of adult ruminants against the colonization by foreign bacterial strains (Weimer et al., 2010), prenatal or early in life modification of microbial communities of the gastrointestinal tract through nutritional interventions has been proposed to improve animal production (Abecia et al., 2013; Jami et al., 2014). Furthermore, early life microbial modulation may persist over a longer period and/or increase the resilience of the microbial community against similar perturbations later in life (Abecia et al., 2014, 2018; Debruyne et al., 2018). In this trial, we assessed whether the animal performance, immune status and microbial community of goat kids were influenced by a prenatal treatment (does fed with *L. leucocephala* forage meal) and/or postnatal intervention (supplementation of active yeast to kids). Additionally, we checked whether these effects would extend beyond the duration of the treatments, when the kids were fed *L. leucocephala* forage meal post-weaning.

At the end of the experimental period (20 weeks), both the pre- as well as the postnatal treatments resulted in an increased body weight and average daily gain. For the prenatal treatment, this could be linked to an enhanced feed intake post-weaning, presumably related to an *in utero* exposure through the maternal ingestion of the same feedstuff. This is in line with previous observations in goats where stimulatory effects of prenatal exposure were demonstrated on the feed intake of diets containing *C*. *odorata*. This plant has nutritionally valuable leaves (e.g., CP exceeding 200 g/kg DM) but a strong and repellent smell (Hai et al., 2012, 2013). Later work of the same group (Hai et al., 2016) confirmed the concept of *in utero* learning as feeding behavior of the offspring did not change when *C. odorata* was supplied to the mother goats during the lactation period only. In contrast to *C. odorata*, *L. leucocephala* supplemented in the current study does not contain odorous compounds. Nevertheless, the presence in *L. leucocephala* of the toxic compound mimosine, could impair digestibility, DMI and animal performance.

As mimosine is a plant secondary metabolite, we hypothesized that systemic responses (e.g., immune response) could occur in non-adapted animals, which could impact DMI and growth. As such, prenatal treatment was expected to alleviate this systemic response. However, at week 20, white blood cell concentrations tended to be lowest in non-treated kids (D–K–) and from weaning onward, serum IgG-levels were lower when kids had been treated prenatally. Nevertheless, differences were relatively small and were not thought to be a driving factor of differences in body weight, ADG and DMI. Additionally, the colostrum of *L. leucocephala-*treated mother goats showed a higher IgM concentration than untreated goats and had a considerably higher amount of IgG (Supplementary Table S12). Ruminant newborns receive immunoglobulin through passive transfer from colostrum (Tizard, 2013) and may show retarded growth when colostrum quantity or quality (e.g., immunoglobulin concentration) is too low (Robison et al., 1988; Elsohaby et al., 2019). In the current study, kids did not differ in BW at birth and 4 weeks of age, but D+ kids were heavier throughout the rest of the experimental period, although at 20 weeks of age an additive effect of yeast supplementation was observed.

Furthermore, the enhanced DMI and/or ADG observed in the D+ animals after weaning, could have been related to the trend of enhanced digestibility of the feed, which was assessed at the end of the experiment. As mimosine impairs rumen degradability (Artiles-Ortega et al., 2021), inclusion of *L. leucocephala* in the does’ diet was hypothesized to enhance the inoculation of mimosine-degrading bacteria in goat kids. *S. jonesii*, the first species that had been identified to possess mimosine-degrading properties, belonging to the phylum Synergistetes, was present in all goat kids in low quantities (less than 1% of relative abundance), which is in line with previous reports (Jami et al., 2013; Wang et al., 2016; Wang L. et al., 2019). Strikingly, at 20 weeks of age, the highest absolute abundance of *S. jonesii* (based on qPCR) was obtained in D– kids, which is hard to explain biologically. However, this treatment effect has not been consistently observed throughout the former samplings, while also the biological relevance of the difference (0.1 log units) seems minor. Meanwhile, other mimosine-degrading species have been identified [*Streptococcus lutetiensis*, *Clostridium butyricum*, and *Lactobacillus vitulinus* (Dominguez-Bello and Stewart, 1991; Derakhshani et al., 2016)]. No qPCR data have been generated for these species. The metataxonomic analysis indicated the genera *Streptococcus* and *Lactobacillus* to be present in low quantities in all goat kids (less than 1% of relative abundance) without differences between treatment groups. In addition, the genus *Clostridium* decreased with age irrespective of the prenatal treatment. Nevertheless, during the second month, the highest relative abundance was observed in D+K+ kids. Hence, it seems unlikely that feeding of *L. leucocephala* during pregnancy has enhanced the inoculation and proliferation of mimosine-degrading bacteria in the offspring. As such, changes in these bacteria could not be linked to variation in DMI and ADG, or digestibility of the diets. Other authors have suggested dietary supplementation of *L. leucocephala* to increase cellulolytic and proteolytic bacteria while reducing the protozoa population (Galindo et al., 2005, 2007, 2009). qPCR data of the current study did not suggest consistent changes in protozoal numbers in D+ kids, nor in representative key cellulolytic bacteria (*Fibrobacter succinogenes*, *R. albus*, and *R. flavefaciens*), while, at the age of 20 weeks the relative abundance of *Ruminococcus* increased in treated kids. The genus *Ruminococcus* includes important fiber degrading species, which are one of the early colonizers in the rumen (Koike and Kobayashi, 2001; Denman and McSweeney, 2006). As such, it is not surprising to observe a prenatal effect in this bacterial genus.

The rumen bacterial richness and diversity increased with age irrespective of pre- or postnatal treatments. Furthermore, there were changes in bacterial composition which linked to the animal’s age. This is in line with Furman et al. (2020), who demonstrated that age globally affects bacterial composition independent of diet. As age is the major determinant of the microbial composition particularly during the pre-weaning period, early life treatment effects can be masked by age-related changes. In our study, the postnatal effects on bacterial composition were more obvious after weaning (14 and 20 weeks), once the rumen fully developed and the bacterial composition was more stable. At phylum level, the importance of Bacteroidetes is known to increase with age, while Firmicutes are decreasing (Jami et al., 2013; Abecia et al., 2017). This development could be accelerated by yeast supplementation immediately after birth. Recent work showed that yeast fed to cattle early in life enhanced bacterial diversity in the rumen, which persisted throughout the trial (Newbold and Ramos-Morales, 2020). In our study, Firmicutes (14 weeks) and Proteobacteria (20 weeks) tended to be less abundant while the relative abundance of the genus *Prevotella* (phylum Bacteroidetes) tended to be higher at 20 weeks in K+ kids, which is in line with the observations reported by Newbold and Ramos-Morales (2020) and Peng et al. (2020). According to AlZahal et al. (2017), yeast supplementation did not affect the family Ruminococcaceae. However, in the current study, the relative abundance of Uncul_Ruminococcaceae (the most abundant genus in the Ruminococcaceae family) decreased in K+ kids at 14 weeks, with a concomitant increase of the genus BS11. A similar observation was reported by Welty et al. (2019), who suggested that both families possibly occupy the same niche. Additionally, the core Uncul_Lachnospiraceae and Uncul_Ruminococcaceae (the most abundant genus in the Lachnospiraceae and Ruminococcaceae families, respectively) were higher in K+ kids 12 weeks after the yeast supplementation ceased, while there were no differences in these genera in the overall microbiome at this age.

Furthermore, the qPCR analysis showed protozoa remained absent in the rumen at 4 and 8 weeks of age in this trial, which may be linked to the removal of the kids from their mother after colostrum ingestion. Similar observations were reported previously in goats raised with milk replacers (Abecia et al., 2017; Debruyne et al., 2018; Belanche et al., 2019). However, *S. cerevisiae* supplementation to K+ kids enhanced the protozoal abundance, similar to the observations by Miranda et al. (1996), while earlier colonization of protozoa took place in the rumen of yeast supplemented lambs (Chaucheyras-Durand and Fonty, 2002). This suggests that yeast supplementation favored the maturation of the rumen microbial ecosystem, which is in line with Chaucheyras-Durand et al. (2019). Furthermore, yeast supplementation favors fibrolytic bacteria (Pinloche et al., 2013; Chaucheyras-Durand et al., 2019; Peng et al., 2020). In this sense, it was observed that *F. succinogenes* and *R. albus* tended to be higher in K+ kids compared to K– kids at 4 weeks, which suggests that colonization of these species was favored by yeast supplementation. We also observed an increased abundance of *S. ruminantium* in K+ kids at 20 weeks, in line with earlier reports in adult ruminants (Pinloche et al., 2013). *Fibrobacter succinogenes*, *R. albus*, and *R. flavefaciens* were quantified to assess the effect on cellulolytic bacteria, since these species are presently recognized as the major cellulolytic bacterial species found in the rumen (Koike and Kobayashi, 2001), whereas *S. ruminantium* was quantified as a representative rumen amylolytic bacterial species (Mackie et al., 1978) and *S. jonesii* was quantified as a major L-mimosine and DHP degrading species found in the rumen (McSweeney et al., 2019).

Yeast supplementation concomitantly enhanced animal performance: ADG and FCE were improved in K+ kids at 4 weeks of age, which persisted (or tended to persist) after weaning. As a result, the BW was higher in these kids. Such persistent effects of yeast supplementation had been observed before by Chaucheyras-Durand and Fonty (2001). Chaucheyras-Durand et al. (2019) suggested a link between the enhanced performance and the improvement of the microbial colonization in the maturing rumen through the use of yeast as a feed additive. They suggested the development of a microbial ecosystem toward a more efficient fiber degradation. In line with these suggestions, enhanced dry matter and organic matter digestibility by K+ kids compared to K– kids has been observed in our study.

## Conclusion

Overall, our findings show predominant age-related changes during the bacterial colonization, which could have masked prenatal and early life treatment effects on the ruminal bacterial composition. Nevertheless, such effects became more evident after weaning. The current study supports the stimulatory effects of prenatal exposure and post-natal supplementation of yeast on the intake of a *L. leucocephala* supplemented diet, reflected in an improved ADG and BW. Postnatal supplementation of yeast favored maturation of the rumen bacterial ecosystem (i.e., greater importance of Bacteroidetes, in particular *Prevotella*, and reduced abundance of Firmicutes) and protozoa colonization. Concomitantly, animal performance parameters and DM and OM digestibility were enhanced even post-weaning, when the supplementation was ceased, suggesting effects of the early-life intervention persisted later in life.

## Data Availability Statement

The data is publicly available at: https://www.ncbi.nlm.nih.gov/bioproject/PRJNA757729.

## Ethics Statement

The animal study was reviewed and approved by Ethical commission of the Faculty of Veterinary Medicine, Ghent University, Belgium (approval number EC2015/12), respectively, following the European Directive (EU) No 241/2014. Written informed consent was obtained from the owners for the participation of their animals in this study.

## Author Contributions

VF, RL-O, and EA-O conceived and designed the experiments. EA-O and BR-B conducted the *in vivo* experiment. EA-O, OP, and JJ performed the bacterial analysis. PF-R and EA-O performed the immunological analysis. EA-O performed the statistical analysis, interpreted the data, and wrote the manuscript. JJ, RL-O, and VF corrected the manuscript and jointly supervised all this work. All authors have read and agreed to the published version of the manuscript and to be accountable for all aspects of the work.

## Conflict of Interest

The authors declare that the research was conducted in the absence of any commercial or financial relationships that could be construed as a potential conflict of interest.

## Publisher’s Note

All claims expressed in this article are solely those of the authors and do not necessarily represent those of their affiliated organizations, or those of the publisher, the editors and the reviewers. Any product that may be evaluated in this article, or claim that may be made by its manufacturer, is not guaranteed or endorsed by the publisher.

## Footnotes

1
https://support.illumina.com
