# Supplementary material for: Performance, Rumen Microbial Community and Immune Status of Goat Kids Fed Leucaena leucocephala Post-weaning as Affected by Prenatal and Early Life Nutritional Interventions
Source: Front Microbiol. 2022 Feb 16;12:769438. doi: 10.3389/fmicb.2021.769438 (PMC8889121; doi:10.3389/fmicb.2021.769438)
Supplement: Supplementary file 1 [file Table_1.DOCX]

Table S1. Feedstuffs and chemical composition of diets daily ingested by pregnant goats during the last 7 weeks of their pregnancy (Prenatal treatment).

|  | **LL supplemented group** | **Control group** |
| --- | --- | --- |
| **Feedstuffs (kg DM/d)** |  |  |
| Commercial concentrate ^A^ | 0.24 | 0.29 |
| Star grass (*Cynodon nlemfuensis*) hay ^B^ | 1.00 | 1.00 |
| *L. leucocephala* forage meal ^C^ | 0.03 | - |
| Mineral salt | 0.01 | 0.01 |
| Vegetable oil | 0.01 | - |
| **Chemical composition (g/kg DM)** | | |
| CP* | 102 | 100 |
| NDF* | 609 | 612 |
| ADF* | 295 | 296 |
| Ca | 5.6 | 5.3 |
| P | 3.5 | 3.5 |
| ME* (MJ/kg DM) | 8.8 | 8.7 |

^A^ Ingredients (g/kg): corn grain (422), wheat bran (300), soybean meal (228), calcium salts (40), NaCl (5), and trace minerals and vitamins supplement (4.6). Chemical composition (g/kg DM): OM (945), CP (160), NDF (178), ADF (67.5), ADL (16.7), Ether extract (29.4), Starch (415), Glucose (21.0), ME (11.6 MJ/kg DM) ([Lima-Orozco et al., 2015](#_ENREF_8)).

^B^ Chemical composition (g/kg DM): CP (90), NDF (693), ADF (337), Ca (4), P (2), and ME (8.2 MJ/kg DM).

^C^ Chemical composition (g/kg DM): CP (297), NDF (582), ADF (347), ADL (105), and ME (9.0 MJ/kg DM).

* Abbreviations: CP (Crude protein), NDF (Neutral detergent fiber), ADF (Acid detergent fiber), ME (metabolizable energy).

Table S2. Proximate chemical composition of feed offered to goat kids from the 2^nd^ day of age until weaning (g/kg DM).

|  | OM* | CP* | CF* | EE* | Ca | P |
| --- | --- | --- | --- | --- | --- | --- |
| Milk replacer (Kalvowin) ^A^ | 925 | 212 | 11 | 220 | 4 | 6 |
| Lacto pre-starter (Raltec TC-01) ^B^ | 933 | 166 | 45 | 46 | 9 | 6 |

^A^ Composition: Lactoserum powder, Vegetable oils, Soybean GMO protein concentrated, Soy GMO flour, Lactoserum protein concentrated, Lactose-free whey flour, Vitamins, Mineral salts.

^B^ Composition: Corn, Barley, Roasted and husked soybean flour, Wheat, Vegetable oils, Lactoserum powder, Bicalcium phosphate, Calcium carbonate, Sodium chloride, Wheat flour.

* Abbreviations: OM (Organic Matter), CP (Crude protein), CF (Crude fiber), EE (Ether extract).

Table S3. Calculation of the required minimum sample size per treatment group needed to study different parameters targeted in our study, based on literature data. In all cases, the test family was a factorial ANOVA.

| Parameter | Reported averages per groups | n^3^ | Standard Deviation (pooled) | N_Group_^4^ | Treatment applied | α error^1^ | Power^2^ | Reference | Min Sample size |
| --- | --- | --- | --- | --- | --- | --- | --- | --- | --- |
| Final BW (kg) | 16.1 *vs*. 16.4 *vs*. 16.8 *vs*. 16.2 | 5 | 0.31 | 4 | prenatal and 2 post-natal exposure to *C. odorata* | 0.05 | 0.8 | ([Hai et al., 2013](#_ENREF_6)) | 5 |
| Final BW (kg) | 9.7 *vs*. 10.7 *vs*. 11.3 *vs*. 12.7 | 8 | 1.25 | 4 | 2 weaning ages (d21 vs d35) and 2 levels of milk replacer (2 vs 4 % of BW) | 0.05 | 0.8 | ([Zhang et al., 2019](#_ENREF_15)) | 4 |
| Weaning BW (kg) | 16.4 *vs*. 16.4 *vs*. 15.3 *vs*. 17.6 | 10 | 0.94 | 4 | prenatal and 2 post-natal exposure to coconut oil | 0.05 | 0.8 | ([Debruyne et al., 2018](#_ENREF_3)) | 7 |
| *Prevotella* (%) | 2.48 *vs*. 1.55 *vs*. 4.15 *vs*. 9.81 | 4 | 0.370 | 4 | 4 dietary treatments (nutrient levels were 84, 96, 108, or 120% of the standard) | 0.05 | 0.8 | ([Wang et al., 2017](#_ENREF_14)) | 3 |
| *Prevotellaceae* (%) | 26 *vs*. 26 *vs*. 35 | 5 | 5.8 | 3 | 2 dietary oil supplements (Control *vs*. linseed oil *vs*. heated linseed) | 0.05 | 0.8 | ([Wang et al., 2019](#_ENREF_13)) | 5 |
| Shannon index | 3.97 *vs*. 3.83 *vs*. 4.10 | 15 | 0.137 | 3 | 3 diets (Control *vs*. linseed oil supplemented *vs*. linseed oil pre weaning only) | 0.05 | 0.8 | ([Lyons et al., 2017](#_ENREF_9)) | 6 |
| Shannon index | 4.03 *vs*. 4.52 *vs*. 4.45 *vs*. 4.28 | 4 | 0.218 | 4 | 4 dietary treatments (nutrient levels were 84, 96, 108, or 120% of the standard) | 0.05 | 0.8 | ([Wang et al., 2017](#_ENREF_14)) | 4 |

^1^The statistical error made in testing a hypothesis when it is concluded that a result is positive, but it really is not. Also known as false positive.

^2^The statistical power ranges from 0 to 1, and as statistical power increases, the probability of making a type II error (wrongly failing to reject the null hypothesis) decreases.

^3^n = number of animals included per group in the cited study

^4^NGroup = number of groups used in the statistical test.

Debruyne, S., Ruiz-González, A., Artiles-Ortega, E., Ampe, B., Van Den Broeck, W., De Keyser, E., Vandaele, L., Goossens, K., and Fievez, V. (2018). Supplementing goat kids with coconut medium chain fatty acids in early life influences growth and rumen papillae development until 4 months after supplementation but effects on in vitro methane emissions and the rumen microbiota are transient. *J Anim Sci* 96**,** 1978-1995. doi: 10.1093/jas/sky070.

Hai, P.V., Schonewille, J.T., Tien, D.V., Everts, H., and Hendriks, W.H. (2013). Improved acceptance of Chromonaela odorata by goat kids after weaning is triggered by in utero exposure but not consumption of milk *Applied Animal Behaviour Science* 146**,** 66-71. doi: 10.1016/j.applanim.2013.03.011.

Lyons, T., Boland, T., Storey, S., and Doyle, E. (2017). Linseed Oil Supplementation of Lambs' Diet in Early Life Leads to Persistent Changes in Rumen Microbiome Structure. *Front Microbiol* 8**,** 1656-1656. doi: 10.3389/fmicb.2017.01656.

Wang, X., Martin, G.B., Wen, Q., Liu, S., Zhang, J., Yu, Y., Shi, B., Guo, X., Zhao, Y., and Yan, S. (2019). Linseed oil and heated linseed grain supplements have different effects on rumen bacterial community structures and fatty acid profiles in cashmere kids1. *J Anim Sci* 97**,** 2099-2113. doi: 10.1093/jas/skz079.

Wang, Y., Cao, P., Wang, L., Zhao, Z., Chen, Y., and Yang, Y. (2017). Bacterial community diversity associated with different levels of dietary nutrition in the rumen of sheep. *Applied Microbiology and Biotechnology* 101**,** 3717-3728. doi: 10.1007/s00253-017-8144-5.

Zhang, Q., Li, C., Niu, X., Zhang, Z., Li, F., and Li, F. (2019). The effects of milk replacer allowance and weaning age on the performance, nutrients digestibility, and ruminal microbiota communities of lambs. *Animal Feed Science and Technology* 257**,** 114263. doi: https://doi.org/10.1016/j.anifeedsci.2019.114263.

Table S4. PCR primers used for all the molecular analyses.

| **Target group** | **Oligo name** | **Sequence (5’→3’)** | **Annealing temp. (ºC)** | **Amplicon length (bp)** | **Reference** |
| --- | --- | --- | --- | --- | --- |
| General bacteria | Gbacteria_Fwd | CGGCAACGAGCGCAACCC | 60 | 130 | [Denman and McSweeney (2006)](#_ENREF_4) |
|  | Gbacteria_Rev | CCATTGTAGCACGTGTGTAGCC |  |  |  |
| Anaerobic fungi (*Neocallimastigales*) | Neo QPCR For | TTGACAATGGATCTCTTGGTTCTC | 60 | 110 | [Edwards et al. (2008)](#_ENREF_5) |
|  | Neo QPCR Rev | GTGCAATATGCGTTCGAAGATT |  |  |  |
| *Fibrobacter succinogenes* | Fsuc_Fwd | GTTCGGAATTACTGGGCGTAAA | 60 | 121 | [Denman and McSweeney (2006)](#_ENREF_4) |
|  | Fsuc_Rev | CGCCTGCCCCTGAACTATC |  |  |  |
| *Ruminococcus flavefaciens* | Rflav_Fwd | CGAACGGAGATAATTTGAGTTTACTTAGG | 60 | 132 |  |
|  | Rflav_Rev | CGGTCTCTGTATGTTATGAGGTATTACC |  |  |  |
| *Ruminococcus albus* | Ra1281f | CCCTAAAAGCAGTCTTAGTTCG | 58 | 175 | [Koike and Kobayashi (2001)](#_ENREF_7) |
|  | Ra1439r | CCTCCTTGCGGTTAGAACA |  |  |  |
| *Synergistes jonesii* | Sj_60F | AGTTACAGGGGGACAACGGA | 60 | 313 | [McSweeney et al. (2019)](#_ENREF_10) |
|  | Sj_449R | CGTCACTCGCTTCTTCCCGC |  |  |  |
| *Selenomonas ruminantium* | SelRum2F | CAATAAGCATTCCGCCTGGG | 60 | 138 | [Stevenson and Weimer (2007)](#_ENREF_12) |
|  | SelRum2R | TTCACTCAATGTCAAGCCCTGG |  |  |  |
| Protozoa | 316f | GCTTTCGWTGGTAGTGTATT | 56 | 223 | [Carberry et al. (2012)](#_ENREF_2) |
|  | 539r | CTTGCCCTCYAATCGTWCT |  |  |  |

Table S5. Mean (standard deviation) of α-diversity indices (Chao1, PD_Whole_tree, Observed OTU, Shannon index and dominance) characterizing the rumen bacterial community structure of goat kids at 4, 8, 14 and 20 weeks of age.

| Alpha diversity indices | Age | | | | *P*-value |
| --- | --- | --- | --- | --- | --- |
|  | 4 weeks | 8 weeks | 14 weeks | 20 weeks |  |
|  |  | | | |  |
| Chao1 | 688^c^ (150.1) | 804^b^ (115.8) | 991^a^ (65.5) | 990^a^ (66.8) | <0.001 |
| PD_Whole_tree | 63.8^b^ (13.03) | 73.3^b^ (9.56) | 89.9^a^ (5.42) | 90.7^a^ (4.75) | <0.001 |
| Observed OTU | 617^b^ (140.0) | 713^b^ (115.6) | 895^a^ (70.3) | 902^a^ (58.0) | <0.001 |
| Shannon index | 5.9^b^ (0.64) | 5.9^b^ (0.679) | 6.4^a^ (0.59) | 6.6^a^ (0.46) | <0.001 |
| Dominance | 0.1^a^ (0.04) | 0.1^ab^ (0.04) | 0.1^ab^ (0.03) | 0.0^b^ (0.03) | 0.021 |

Different letters in the same row (^a, b, c^) indicate significant differences among experimental groups (*P* < 0.05) according to the BH procedure ([Benjamini and Hochberg, 1995](#_ENREF_1)).

Table S6. Mean (standard deviation) of absolute abundance of target rumen microbes determined by real-time PCR (log_10_/mL rumen fluid) of goat kids at 4, 8, 14 and 20 weeks of age.

| Index | Age | | | | *P* – value |
| --- | --- | --- | --- | --- | --- |
|  | 4 weeks | 8 weeks | 14 weeks | 20 weeks |  |
| Total Bacteria | 12.4^b^ (0.21) | 12.6^b^ (0.20) | 13.0^a^ (0.31) | 12.9^a^ (0.15) | <0.001 |
| Fungi ^1^ | 9.7^ab^ (0.28) | 10.0^a^ (0.77) | 7.9^c^ (0.47) | 9.5^b^ (0.35) | <0.001 |
| Protozoa | nd | nd | 10.5^b^ (0.61) | 10.9^a^ (0.50) | <0.001 |
| *F. succinogenes* | 10.0^c^ (0.79) | 10.7^b^ (0.73) | 11.1^a^ (0.55) | 10.5^b^ (0.33) | <0.001 |
| *R. flavefasciens* | 8.8^b^ (1.10) | 8.7^b^ (0.75) | 9.6^a^ (0.87) | 9.5^a^ (0.71) | 0.002 |
| *R. albus* | 8.7^c^ (1.65) | 9.4^bc^ (1.21) | 10.4^a^ (0.63) | 10.0^ab^ (0.44) | <0.001 |
| *S. ruminantium* | 11.6^c^ (0.36) | 11.5^c^ (0.30) | 12.2^a^ (0.37) | 11.9^b^ (0.28) | <0.001 |
| *S. jonessi* | 8.5^b^ (0.74) | 8.8^b^ (0.69) | 9.6^a^ (0.26) | 8.8^b^ (0.45) | <0.001 |

Different letters in the same row (^a, b, c^) indicate significant differences among ages (*P*<0.05) according to the BH procedure ([Benjamini and Hochberg, 1995](#_ENREF_1))

^1^ Fungi were determined based on the order *Neocallimastigales*. nd: not detected.

Table S7. Mean (standard deviation) of animal performance and immune status of goat kids at 4, 8, 14 and 20 weeks of age.

|  | Age | | | | *P*-value |
| --- | --- | --- | --- | --- | --- |
|  | 4 weeks | 8 weeks | 14 weeks | 20 weeks |  |
| BW (kg) | 4.6^d^ (0.67) | 6.9^c^ (0.67) | 10.7^b^ (1.00) | 14.5^a^ (1.43) | <0.001 |
| ADG (g/d) | 78.8^ab^ (18.76) | 76.6^b^ (14.44) | 85.9^a^ (9.22) | 81.2^ab^ (8.74) | 0.019 |
| DMI (g/d) | 138^d^ (20.1) | 196^c^ (18.1) | 304^b^ (20.5) | 390^a^ (33.4) | <0.001 |
| FCE (kg/kg) | 1.9^d^ (0.31) | 2.7^c^ (0.33) | 4.0^b^ (0.28) | 5.0^a^ (0.27) | <0.001 |
| Total WBC (*10^9^/L) | 11.2^b^ (1.10) | 10.4^c^ (0.59) | nd | 12.4^a^ (1.36) | <0.001 |
| Lymphocytes (% ^1^) | 60.1 (6.78) | 63.0 (8.56) | nd | 64.0 (6.46) | 0.144 |
| Neutrophils (%) | 37.9 (7.25) | 33.9 (9.53) | nd | 35.3 (7.27) | 0.383 |
| Eosinophils (%) | 1.8^ab^ (2.88) | 2.6^a^ (2.66) | nd | 0.7^b^ (1.53) | 0.027 |
| Monocytes (%) | 0.3^a^ (0.77) | 0.6^a^ (0.86) | nd | 0.1^b^ (0.24) | 0.004 |
| sIgG (mg/mL) | 16.1^b^ (1.79) | 11.8^c^ (1.93) | nd | 29.0^a^ (1.48) | <0.001 |
| mIgG (µg/mL) | 59.1^a^ (22.56) | 20.6^c^ (6.47) | nd | 27.2^b^ (17.09) | <0.001 |
| sIgM (mg/mL) | 0.6^c^ (0.18) | 0.8^b^ (0.08) | nd | 1.4^a^ (0.35) | <0.001 |
| mIgM (µg/mL) | 45.4^a^ (18.00) | 27.0^b^ (14.79) | nd | 11.8^c^ (4.85) | <0.001 |
| ChT (mU/mL) | 244^b^ (42.6) | 255^b^ (48.4) | nd | 280^a^ (46.7) | 0.001 |

Different letters in the same row (^a, b, c, d^) indicate significant differences among experimental groups (*P*<0.05) according to the BH procedure ([Benjamini and Hochberg, 1995](#_ENREF_1)).

^1^ % based on the total blood cells counted.

Abbreviations: BW: body weight; ADG: average daily gain; VFI: voluntary feed intake; FCE: feed conversion efficiency; WBC: white blood cells; Ig: immunoglobulin; s: serum; m: mucosal (saliva); ChT: chitotriosidase activity; nd: not determined.

Table S8. Mean (standard deviation) of relative abundance of rumen bacterial (%) phyla (in **bold**) and their predominant genera (relative abundance > 1 %) of goat kids at 4, 8, 14 and 20 weeks of age.

| Taxa | Treatments | | | | *P*-value | | |
| --- | --- | --- | --- | --- | --- | --- | --- |
|  | D+K+ | D+K- | D-K+ | D-K- | Prenatal | Postnatal | Interaction |
| 4 weeks of age | | | | | | | |
| **Bacteroidetes** | 29.1 (7.80) | 24.8 (12.72) | 19.9 (2.71) | 22.5 (24.93) | 0.361 | 0.940 | 0.901 |
| Uncul_Bacteroidales | 12.7 (13.28) | 16.9 (9.54) | 8.4 (8.60) | 17.9 (22.58) | 0.610 | 0.071 | 0.751 |
| BS11 | 9.4 (11.05) | 1.2 (1.23) | 3.00 (1.87) | 1.2 (1.25) | 0.266 | 0.118 | 0.245 |
| *Prevotella* | 6.5 (7.82) | 6.2 (5.74) | 7.4 (7.05) | 3.0 (1.85) | 0.972 | 0.883 | 0.800 |
| **Firmicutes** | 66.2 (10.44) | 69.0 (14.3) | 76.6 (4.58) | 74.5 (24.82) | 0.387 | 0.766 | 0.999 |
| Uncul_Bacillaceae | 2.7 (1.97) | 8.8 (10.79) | 3.44 (2.94) | 3.4 (2.45) | 0.580 | 0.239 | 0.451 |
| *Bacillus* | 2.4 (2.05) | 3.7 (3.37) | 2.9 (1.67) | 2.8 (1.08) | 0.730 | 0.706 | 0.743 |
| *Solibacillus* | 13.3 (19.52) | 8.7 (6.45) | 8.2 (5.09) | 4.0 (2.31) | 0.963 | 0.834 | 0.491 |
| *Enterococcus* | 1.1 (0.89) | 0.6 (0.52) | 1.7 (2.68) | 2.8 (4.10) | 0.680 | 0.961 | 0.504 |
| *Lactococcus* | 0.1 (0.13) | 0.1 (0.08) | 3.5 (6.93) | 0.7 (1.24) | 0.426 | 0.083 | 0.052 |
| Uncul_Clostridiales | 19.0 (10.80) | 21.6 (6.93) | 21.5 (5.43) | 38.1 (22.28) | 0.224 | 0.213 | 0.284 |
| Uncul_Christensenellaceae | 2.2 (2.95) | 2.2 (1.21) | 3.2 (4.12) | 1.2 (1.07) | 0.708 | 0.947 | 0.500 |
| *Clostridium* | 4.6 (6.13) | 1.0 (2.15) | 5.4 (9.92) | 1.4 (2.32) | 0.816 | 0.308 | 0.871 |
| *Dehalobacterium* | 1.4 (2.75) | 0.3 (0.09) | 0.1 (0.08) | 0.1 (0.10) | 0.429 | 0.483 | 0.522 |
| Uncul_Lachnospiraceae | 2.8 (2.32) | 0.8 (1.43) | 4.1 (2.42) | 3.5 (2.00) | 0.073 | 0.025 | 0.166 |
| Uncul_Ruminococcaceae | 9.9 (6.18) | 14.2 (7.33) | 14.9 (7.98) | 9.3 (6.69) | 0.833 | 0.840 | 0.084 |
| *Ruminococcus* | 1.6 (0.76) | 2.6 (1.73) | 2.4 (0.86) | 2.4 (0.74) | 0.808 | 0.296 | 0.352 |
| **Planctomycetes** (Uncul_Pirellulaceae*) | 1.5 (1.49) | 1.7 (1.39) | 0.8 (0.85) | 0.7 (0.37) | 0.221 | 0.642 | 0.665 |
| **TM7** (F16*) | 2.0 (3.98) | 3.2 (4.15) | 0.1 (0.22) | 0.3 (0.45) | 0.139 | 0.141 | 0.202 |

* In the current microbial dataset, only one genus was detected within the Phyla Plantomycetes and TM7 (Uncul_Pirellulaceae and F16, respectively).

.

Table S8. Continued

| Taxa | Treatments | | | | *P*-value | | |
| --- | --- | --- | --- | --- | --- | --- | --- |
|  | D+K+ | D+K- | D-K+ | D-K- | Prenatal | Postnatal | Interaction |
| 8 weeks of age | | | | | | | |
| **Bacteroidetes** | 17.1 (8.26) | 13.6 (10.26) | 23.1 (17.91) | 10.1 (4.87) | 0.759 | 0.208 | 0.328 |
| Uncul_Bacteroidales | 9.4 (6.87) | 6.4 (4.73) | 9.8 (9.75) | 3.1 (1.98) | 0.814 | 0.144 | 0.548 |
| BS11 | 2.6 (3.04) | 0.4 (0.53) | 0.7 (0.83) | 0.5 (0.30) | 0.142 | 0.073 | 0.215 |
| *Prevotella* | 4.5 (3.47) | 5.9 (8.35) | 12.2 (9.38) | 5.27 (3.20) | 0.275 | 0.435 | 0.291 |
| **Firmicutes** | 69.7 (15.05) | 75.7 (9.57) | 53.9 (17.65) | 68.6 (20.58) | 0.219 | 0.135 | 0.515 |
| Uncul_Bacillaceae | 6.2 (1.97) | 2.0 (10.79) | 4.0 (2.94) | 7.9 (2.45) | 0.661 | 0.730 | 0.180 |
| *Bacillus* | 2.2 (1.91) | 2.0 (2.53) | 1.0 (1.05) | 1.8 (1.83) | 0.534 | 0.997 | 0.561 |
| *Solibacillus* | 5.1 (19.52) | 12.6 (6.45) | 8.9 (5.09) | 3.0 (2.31) | 0.531 | 0.634 | 0.315 |
| *Enterococcus* | 0.2 (0.15) | 0.65 (1.06) | 4.8 (9.33) | 0.7 (0.79) | 0.447 | 0.529 | 0.269 |
| *Lactococcus* | 0.0 (0.00) | 6.1 (13.69) | 0.0 (0E+00) | 0.1 (0.16) | 0.386 | 0.484 | 0.361 |
| Uncul_Clostridiales | 19.8 (12.53) | 19.8 (10.26) | 16.0 (11.59) | 13.7 (3.89) | 0.387 | 0.509 | 0.606 |
| *Clostridium* | 1.2 (1.23) | 0.8 (0.94) | 0.1 (0.06) | 0.1 (0.25) | 0.002 | 0.468 | 0.226 |
| Uncul_Lachnospiraceae | 4.3 (2.38) | 2.6 (1.55) | 5.6 (3.41) | 3.4 (0.62) | 0.487 | 0.187 | 0.909 |
| *Butyrivibrio* | 1.1 (1.41) | 2.4 (4.40) | 3.5 (2.99) | 8.8 (9.25) | 0.261 | 0.051 | 0.213 |
| Uncul_Ruminococcaceae | 19.0 (14.42) | 17.5 (12.95) | 2.9 (2.10) | 13.9 (19.21) | 0.206 | 0.993 | 0.484 |
| *Ruminococcus* | 6.3 (5.21) | 3.5 (3.88) | 3.7 (2.25) | 9.5 (8.17) | 0.527 | 0.738 | 0.081 |
| **Planctomycetes** (Uncul_Pirellulaceae*) | 5.4 (5.53) | 1.7 (1.91) | 2.5 (2.93) | 2.0 (2.89) | 0.531 | 0.201 | 0.567 |
| **Proteobacteria** | 4.4 (8.13) | 4.7 (4.27) | 19.0 (19.86) | 14.2 (20.15) | 0.246 | 0.890 | 0.592 |
| Uncul_Moraxellaceae | 3.0 (6.52) | 2.9 (3.73) | 17.2 (17.70) | 13.3 (19.17) | 0.108 | 0.829 | 0.514 |
| *Acinetobacter* | 0.2 (0.34) | 1.4 (3.03) | 0.1 (0.11) | 0.0 (0.02) | 0.412 | 0.300 | 0.376 |
| *Psychrobacter* | 0.8 (1.04) | 0.3 (0.33) | 1.7 (2.31) | 0.8 (1.03) | 0.423 | 0.651 | 0.899 |
| **Spirochaetes** | 1.2 (1.31) | 0.5 (1.0) | 0.1 (0.19) | 0.4 (0.66) | 0.207 | 0.392 | 0.320 |
| *Treponema* | 1.2 (1.33) | 0.5 (1.02) | 0.1 (0.19) | 0.3 (0.55) | 0.240 | 0.358 | 0.321 |
| **TM7** (F16*) | 0.8 (0.78) | 2.6 (3.41) | 0.6 (0.56) | 3.5 (3.75) | 0.579 | 0.150 | 0.420 |

* In the current microbial dataset, only one genus was detected within the Phyla Plantomycetes and TM7 (Uncul_Pirellulaceae and F16, respectively).

Table S8. Continued

| Taxa | Treatments | | | | *P*-value | | |
| --- | --- | --- | --- | --- | --- | --- | --- |
|  | D+K+ | D+K- | D-K+ | D-K- | Prenatal | Postnatal | Interaction |
| 14 weeks of age | | | | | | | |
| **Bacteroidetes** | 37.8 (7.49) | 38.5 (13.80) | 47.9 (2.68) | 31.8 (14.88) | > 0.999 | 0.270 | 0.129 |
| Uncul_Bacteroidales | 12.8 (4.29) | 18.1 (8.32) | 14.6 (1.97) | 21.0 (11.53) | 0.313 | 0.173 | 0.996 |
| BS11 | 13.0 (4.75) | 2.9 (2.94) | 14.8 (3.20) | 4.8 (8.17) | 0.503 | 0.002 | 0.313 |
| *Prevotella* | 11.4^bc^ (5.65) | 15.9^ab^ (6.40) | 17.3^a^ (4.11) | 3.9^c^ (2.63) | 0.215 | 0.175 | 0.004 |
| **Firmicutes** | 44.9 (5.21) | 54.2 (11.64) | 45.3 (2.13) | 55.8 (18.22) | 0.826 | 0.073 | 0.565 |
| *Planomicrobium* | 3.8 (5.23) | 0.3 (0.45) | 0.2 (0.18) | 0.9 (1.79) | 0.364 | 0.364 | 0.189 |
| *Sporosarcina* | 0.0 (0.07) | 3.8 (7.28) | 0.5 (0.91) | 8.3 (16.61) | 0.574 | 0.236 | 0.574 |
| *Desemzia* | 9.1 (11.10) | 0.2 (0.50) | 5.8 (5.78) | 4.0 (8.01) | 0.813 | 0.072 | 0.966 |
| Uncul_Clostridiales | 12.6^b^ (3.44) | 18.5^ab^ (6.83) | 18.1^a^ (1.64) | 14.6^ab^ (3.98) | 0.394 | 0.848 | 0.004 |
| Uncul_Christensenellaceae | 0.5 (0.35) | 2.3 (2.59) | 0.8 (0.45) | 0.9 (0.87) | 0.455 | 0.104 | 0.246 |
| Uncul_Lachnospiraceae | 6.2^ab^ (3.09) | 3.0^b^ (0.85) | 3.1^b^ (1.10) | 7.6^a^ (4.62) | 0.643 | 0.831 | 0.049 |
| *Butirivibrio* | 1.3 (0.36) | 1.7 (1.51) | 2.4 (1.87) | 1.3 (0.04) | 0.394 | 0.493 | 0.346 |
| Uncul_Ruminococcaceae | 4.9 (2.22) | 16.3 (10.38) | 5.2 (1.25) | 12.9 (10.28) | 0.347 | 0.002 | 0.399 |
| *Ruminococcus* | 4.5 (2.88) | 4.3 (2.22) | 6.9 (6.67) | 2.5 (1.48) | 0.697 | 0.459 | 0.319 |
| **Planctomycetes** (Uncul_Pirellulaceae*) | 4.4 (3.54) | 1.2 (1.06) | 2.3 (1.81) | 3.0 (3.12) | 0.588 | 0.230 | 0.287 |
| **Proteobacteria** | 7.2 (15.70) | 0.2 (0.05) | 0.0 (0.02) | 1.1 (1.15) | 0.403 | 0.480 | 0.372 |
| Uncul_Moraxellaceae | 7.0 (15.70) | 0.0 (0.02) | 0.0 (0.00) | 0.5 (0.93) | 0.383 | 0.425 | 0.358 |
| **Spirochaetes** (*Treponema**) | 2.1 (0.96) | 3.1 (1.97) | 1.4 (1.23) | 1.6 (1.3) | 0.188 | 0.400 | 0.593 |
| **TM7** (F16*) | 2.8 (1.92) | 2.1 (2.23) | 2.2 (0.45) | 5.3 (5.67) | 0.463 | 0.777 | 0.223 |

Different letters in the same row (^a, b, c^) indicate significant differences among treatments (P<0.05) according to the BH procedure ([Benjamini and Hochberg, 1995](file:///E:/Doctorado/Experimental%20Early%20life%20programming/Papers/2nd%20paper/Table_Results.docx#_ENREF_5)).

* In the current microbial dataset, only one genus was detected within the Phyla Plantomycetes, Spirochaetes and TM7 (Uncul_Pirellulaceae, *Treponema* and F16, respectively)..

Table S8. Continued.

| Taxa | Treatments | | | | *P*-value | | |
| --- | --- | --- | --- | --- | --- | --- | --- |
|  | D+K+ | D+K- | D-K+ | D-K- | Prenatal | Postnatal | Interaction |
| 20 weeks of age | | | | | | | |
| **Bacteroidetes** | 37.8 (6.03) | 37.0 (12.77) | 46.2 (11.09) | 41.3 (6.42) | 0.183 | 0.506 | 0.457 |
| Uncul_Bacteroidales | 12.9 (4.62) | 17.9 (12.33) | 24.0 (3.15) | 18.6 (7.59) | 0.139 | 0.918 | 0.263 |
| BS11 | 5.3 (4.83) | 6.2 (5.89) | 1.4 (1.83) | 3.1 (4.82) | 0.156 | 0.845 | 0.993 |
| *Prevotella* | 19.0 (2.91) | 11.6 (8.89) | 20.3 (9.09) | 17.4 (5.07) | 0.244 | 0.070 | 0.306 |
| S24-7 | 0.4 (0.76) | 0.6 (0.59) | 0.0 (0.04) | 1.3 (2.56) | 0.386 | 0.165 | 0.147 |
| **Chloroflexi** (SHD-231*) | 3.2^a^ (2.75) | 0.3^b^ (0.19) | 0.6^ab^ (0.49) | 0.6^ab^ (0.37) | 0.047 | 0.057 | 0.049 |
| **Firmicutes** | 52.0 (5.07) | 50.4 (17.69) | 46.0 (17.62) | 44.9 (7.03) | 0.389 | 0.589 | 0.758 |
| *Planomicrobium* | 1.7 (3.80) | 0.1 (0.33) | 0.1 (0.19) | 0.0 (0.02) | 0.403 | 0.261 | 0.366 |
| *Sporosarcina* | 0.0 (0.05) | 0.0 (0.01) | 0.1 (0.18) | 3.6 (7.24) | 0.337 | 0.464 | 0.464 |
| *Carnobacterium* | 0.0 (0.02) | 6.0 (12.67) | 0.0 (0.00) | 0.0 (0.02) | 0.463 | 0.394 | 0.295 |
| Uncul_Clostridiales | 17.4 (6.32) | 16.9 (6.02) | 20.3 (10.96) | 13.9 (2.80) | 0.759 | 0.251 | 0.416 |
| Uncul_Christensenellaceae | 0.7 (0.39) | 1.9 (1.92) | 0.9 (0.30) | 0.7 (0.40) | 0.325 | 0.054 | 0.116 |
| Uncul_Lachnospiraceae | 7.9 (4.43) | 6.7 (4.14) | 9.2 (1.30) | 8.8 (4.33) | 0.268 | 0.352 | 0.849 |
| *Butyrivibrio* | 4.7 (3.43) | 3.5 (4.22) | 4.6 (1.23) | 4.7 (2.74) | 0.268 | 0.352 | 0.849 |
| Uncul_Ruminococcaceae | 10.3 (1.28) | 9.7 (3.47) | 8.0 (2.07) | 7.1 (1.96) | 0.139 | 0.672 | 0.981 |
| *Ruminococcus* | 6.6 (3.50) | 3.6 (3.24) | 0.8 (0.53) | 2.4 (2.56) | 0.028 | 0.499 | 0.052 |
| **Planctomycetes** (Uncul_Pirellulaceae*) | 2.5 (2.09) | 2.7 (1.40) | 1.4 (0.64) | 4.0 (5.18) | 0.690 | 0.460 | 0.826 |
| **Proteobacteria** | 0.2 (0.13) | 6.9 (14.41) | 0.1 (0.03) | 6.6 (12.22) | 0.575 | 0.079 | 0.857 |
| Uncul_Moraxellaceae | 0.0 (0.02) | 0.1 (0.28) | 0.0 (0.00) | 5.9 (11.85) | 0.306 | 0.276 | 0.184 |
| *Psychrobacter* | 0.1 (0.11) | 6.6 (14.15) | 0.0 (0.02) | 0.3 (0.54) | 0.390 | 0.504 | 0.432 |
| **Spirochaetes** (*Treponema**) | 1.5 (1.07) | 0.5 (0.48) | 1.8 (2.00) | 0.6 (0.46) | 0.865 | 0.160 | 0.942 |
| **TM7** (F16*) | 1.9 (1.57) | 1.7 (0.71) | 3.3 (0.28) | 1.4 (0.33) | 0.272 | 0.037 | 0.063 |

Different letters in the same row (^a, b^) indicate significant differences among treatments (P<0.05) according to the BH procedure ([Benjamini and Hochberg, 1995](file:///E:/Doctorado/Experimental%20Early%20life%20programming/Papers/2nd%20paper/Table_Results.docx#_ENREF_5)).

* In the current microbial dataset, only one genus was detected within the Phyla Chloroflexi, Plantomycetes, Spirochaetes and TM7 (SHD-231, Uncul_Pirellulaceae, Treponema and F16, respectively).

Table S9. Mean (standard deviation) of relative abundance of core-persistent microbiome (phyla [**in bold**] and genus level) which were observed in 80% of the goat kids irrespective of treatment at the age of 4 weeks and which persisted through 8 and 14 weeks of age till 20 weeks of age.

| Taxa | Age | | | | *P*-value |
| --- | --- | --- | --- | --- | --- |
|  | 4 weeks | 8 weeks | 14 weeks | 20 weeks |  |
|  |  | | | |  |
| **Actinobacteria** (Coriobacteriaceae*) | 0.2^a^ (0.30) | 0.1^b^ (0.10) | 0.0^bc^ (0.06) | 0.0^c^ (0.01) | < 0.001 |
| **Bacteroidetes** (Uncul_Bacteroidales*) | 6.4^a^ (8.37) | 1.0^b^ (1.43) | 0.4^b^ (0.34) | 0.4^b^ (0.24) | < 0.001 |
| **Firmicutes** | 13.3 (5.64) | 13.0 (9.48) | 10.5 (3.81) | 13.6 (5.18) | 0.128 |
| Uncul_Clostridiales | 7.3 (3.76) | 6.0 (5.04) | 6.7 (2.37) | 8.4 (4.76) | 0.164 |
| Uncul_Mogibacteriaceae | 0.0^b^ (0.04) | 0.1^a^ (0.12) | 0.0^b^ (0.04) | 0.1^a^ (0.06) | < 0.001 |
| Uncul_Christensenellaceae | 1.1^a^ (1.14) | 0.2^b^ (0.19) | 0.2^b^ (0.12) | 0.5^a^ (0.30) | < 0.001 |
| Uncul_Clostridiaceae | 0.1^b^ (0.10) | 0.2^a^ (0.20) | 0.1^ab^ (0.13) | 0.1^a^ (0.05) | 0.015 |
| Uncul_Lachnospiraceae | 1.3^b^ (1.34) | 1.3^b^ (1.20) | 1.7^ab^ (2.18) | 2.2^a^ (1.24) | 0.045 |
| Uncul_Ruminococcaceae | 3.0^a^ (2.90) | 2.5^ab^ (6.54) | 1.6^b^ (1.30) | 2.1^ab^ (0.98) | 0.005 |
| Oscillospira | 0.0 (0.05) | 0.0 (0.02) | 0.0 (0.02) | 0.0 (0.02) | 0.647 |
| Ruminococcus | 0.5^a^ (0.68) | 2.5^a^ (3.62) | 0.5^a^ (0.64) | 0.1^b^ (0.14) | < 0.001 |
| Other_Ruminococcaceae | 0.0^c^ (0.02) | 0.1^ab^ (0.32) | 0.1^b^ (0.04) | 0.1^a^ (0.09) | < 0.001 |

Different letters in the same row (^a, b, c^) indicate significant differences among ages (*P*<0.05) according to the BH procedure ([Benjamini and Hochberg, 1995](#_ENREF_1)).

* In the current microbial dataset, only one genera was detected within the Phyla Actinobacteria and Bacteroidetes (Coriobacteriaceae and Uncul_Bacteroidales, respectively).

Table S10. Mean (standard deviation) of the animal performance and immune status of goat kids at 4, 8, 14 and 20 weeks of age.

| Index (Units) | Treatments | | | | *P*-value | | |
| --- | --- | --- | --- | --- | --- | --- | --- |
|  | D+K+ | D+K- | D-K+ | D-K- | Prenatal | Postnatal | Interaction |
|  |  |  |  |  |  |  |  |
| BW 0d (kg) | 2.4 (0.38) | 2.4 (0.38) | 2.2 (0.15) | 2.1 (0.04) | 0.443 | - | - |
|  | 4 weeks | | | |  |  |  |
| BW (kg) | 5.4 (0.61) | 4.6 (0.54) | 4.4 (0.48) | 4.3 (0.58) | 0.106 | 0.063 | 0.164 |
| ADG (g/d) | 99.8^a^ (14.15) | 73.3^b^ (16.28) | 72.4^b^ (14.39) | 74.9^b^ (18.30) | 0.221 | 0.041 | 0.050 |
| DMI (g/d) | 149 (20.9) | 142 (19.2) | 121 (17.4) | 137 (14.9) | 0.162 | 0.814 | 0.148 |
| FCE (kg/kg) | 1.6 (0.19) | 2.1 (0.33) | 1.8 (0.17) | 2.0 (0.30) | 0.597 | 0.001 | 0.336 |
| Total WBC (*10^9^/L) | 11.3 (1.08) | 11.6 (1.54) | 11.0 (0.87) | 10.5 (0.49) | 0.479 | 0.611 | 0.531 |
| Lymphocytes (% ^1^) | 62.2 (8.67) | 59.6 (8.59) | 58.5 (3.79) | 59.3 (5.51) | 0.607 | 0.867 | 0.680 |
| Neutrophils (%) | 37.0 (8.12) | 36.6 (10.73) | 39.5 (3.00) | 39.4 (5.57) | 0.542 | 0.748 | 0.950 |
| Eosinophils (%) | 0.8 (1.10) | 3.6 (4.83) | 1.00 (1.41) | 1.3 (1.53) | 0.432 | 0.146 | 0.537 |
| Monocytes (%) | 0.0^b^ (0E+00) | 0.2^ab^ (0.45) | 1.00^a^ (1.41) | 0.0^b^ (0E+00) | 0.251 | 0.262 | 0.049 |
| sIgG (mg/mL) | 17.3^a^ (1.45) | 15.0^b^ (0.43) | 14.4^b^ (1.06) | 17.9^a^ (1.46) | 0.939 | 0.760 | 0.001 |
| mIgG (µg/mL) | 39.3^b^ (5.71) | 90.3^a^ (11.32) | 54.1^a^ (5.31) | 47.0^b^ (4.48) | 0.006 | <0.001 | <0.001 |
| sIgM (mg/mL) | 0.8^a^ (0.08) | 0.6^b^ (0.03) | 0.4^c^ (0.04) | 0.4^c^ (0.03) | 0.002 | 0.003 | 0.001 |
| mIgM (µg/mL) | 34.9^b^ (7.12) | 57.5^ab^ (27.93) | 50.9^a^ (4.77) | 35.3^b^ (6.25) | 0.940 | 0.268 | 0.012 |
| ChT (mU/mL) | 275^a^ (18.5) | 194^b^ (19.0) | 232^b^ (16.2) | 291^a^ (21.0) | 0.049 | 0.057 | <0.001 |

Table S10. Continued

| Index | Treatments ^1^ | | | | *P*-value ^2^ | | |
| --- | --- | --- | --- | --- | --- | --- | --- |
|  | D+K+ | D+K- | D-K+ | D-K- | Prenatal | Postnatal | Interaction |
|  |  |  |  |  |  |  |  |
|  | 8 weeks | | | |  |  |  |
| BW (kg) | 7.4 (0.53) | 7.2 (0.57) | 6.5 (0.71) | 6.6 (0.66) | 0.045 | 0.458 | 0.771 |
| ADG (g/d) | 80.4 (12.43) | 80.2 (20.51) | 70.5 (13.42) | 78.7 (11.93) | 0.433 | 0.625 | 0.586 |
| DMI (g/d) | 203 (17.3) | 201 (20.1) | 177 (13.7) | 200 (10.6) | 0.208 | 0.114 | 0.055 |
| FCE (kg/kg) | 2.6 (0.34) | 2.7 (0.23) | 2.7 (0.49) | 2.9 (0.27) | 0.398 | 0.119 | 0.513 |
| Total WBC (*10^9^/L) | 10.8 (0.45) | 10.2 (0.37) | 10.7 (0.72) | 9.9 (0.20) | 0.555 | 0.005 | 0.934 |
| Lymphocytes (% ^1^) | 64.0 (8.28) | 64.2 (11.35) | 60.5 (4.65) | 62.8 (10.72) | 0.658 | 0.789 | 0.819 |
| Neutrophils (%) | 32.4 (10.17) | 33.0 (10.67) | 36.0 (8.79) | 34.8 (11.53) | 0.501 | 0.934 | 0.925 |
| Eosinophils (%) | 3.2 (3.49) | 2.0 (1E+00) | 2.8 (4.27) | 2.3 (1.71) | 0.816 | 0.762 | 0.761 |
| Monocytes (%) | 0.4 (0.55) | 0.8 (0.84) | 0.8 (1.50) | 0.3 (0.50) | 0.612 | 0.585 | 0.538 |
| sIgG (mg/mL) | 11.2 (0.79) | 9.4 (0.41) | 14.3 (0.36) | 12.3 (0.87) | <0.001 | <0.001 | 0.973 |
| mIgG (µg/mL) | 21.1^ab^ (2.35) | 28.6^a^ (3.27) | 16.2^b^ (4.11) | 14.2^b^ (1.63) | <0.001 | 0.046 | 0.007 |
| sIgM (mg/mL) | 0.9 (0.07) | 0.8 (0.04) | 0.9 (0.01) | 0.8 (0.12) | 0.550 | 0.025 | 0.703 |
| mIgM (µg/mL) | 45.9 (14.41) | 22.7 (7.09) | 20.8 (2.22) | 15.1 (6.40) | 0.003 | 0.010 | 0.075 |
| ChT (mU/mL) | 268^a^ (46.3) | 230^ab^ (38.4) | 216^b^ (34.2) | 308^a^ (16.6) | 0.243 | 0.199 | 0.008 |

.

Table S10. Continued

| Index | Treatments | | | | *P*-value | | | |
| --- | --- | --- | --- | --- | --- | --- | --- | --- |
|  | D+K+ | D+K- | D-K+ | D-K- | Prenatal | Postnatal | | Interaction |
|  |  | | | |  |  | |  |
|  | 14 weeks | | | |  |  | |  |
| BW (kg) | 11.7 (0.71) | 10.8 (1.00) | 10.4 (0.78) | 9.9 (0.58) | 0.025 | 0.068 | | 0.927 |
| ADG (g/d) | 95.7 (7.16) | 85.7 (9.27) | 83.8 (8.67) | 81.5 (6.34) | 0.129 | 0.105 | | 0.610 |
| DMI (g/d) | 314 (17.0) | 311 (24.2) | 286 (15.2) | 301 (16.6) | 0.073 | | 0.523 | 0.203 |
| FCE (kg/kg) | 3.7 (0.16) | 4.1 (0.15) | 3.8 (0.25) | 4.3 (0.14) | 0.151 | 0.001 | | 0.319 |
|  | 20 weeks | | | |  |  | |  |
| BW (kg) | 16.2 (1.00) | 14.1 (1.16) | 14.5 (1.00) | 13.2 (0.48) | 0.024 | 0.006 | | 0.456 |
| ADG (g/d) | 92.4 (5.89) | 78.5 (6.95) | 81.6 (6.99) | 75.6 (4.31) | 0.046 | 0.004 | | 0.445 |
| DMI (g/d) | 418 (25.8) | 393 (36.7) | 371 (26.4) | 368 (22.3) | 0.050 | | 0.135 | 0.761 |
| FCE (kg/kg) | 4.8 (0.10) | 5.3 (0.13) | 4.8 (0.12) | 5.2 (0.14) | 0.914 | <0.001 | | 0.945 |
| Total WBC (*10^9^/L) | 13.3 (0.65) | 12.4 (1.48) | 13.0 (0.95) | 10.7 (0.40) | 0.026 | 0.003 | | 0.092 |
| Lymphocytes (% ^1^) | 64.6 (5.73) | 65.6 (6.80) | 63.3 (5.38) | 66.3 (9.74) | 0.939 | 0.400 | | 0.700 |
| Neutrophils (%) | 35.4 (8.88) | 33.6 (6.35) | 36.8 (5.38) | 31.3 (7.37) | 0.442 | 0.156 | | 0.973 |
| Eosinophils (%) | 0.0^b^ (0E+00) | 0.8^ab^ (1.79) | 0.0^b^ (0E+00) | 2.3^a^ (2.06) | 0.042 | 0.005 | | 0.049 |
| Monocytes (%) | 0.0 (0E+00) | 0.0 (0E+00) | 0.0 (0E+00) | 0.3 (0.50) | 0.245 | 0.201 | | 0.165 |
| sIgG (mg/mL) | 28.9 (0.64) | 27.8 (1.40) | 30.8 (1.88) | 28.9 (0.91) | 0.039 | 0.038 | | 0.298 |
| mIgG (µg/mL) | 15.1^c^ (4.99) | 23.0^b^ (1.92) | 17.4^c^ (1.86) | 57.3^a^ (1.76) | <0.001 | <0.001 | | <0.001 |
| sIgM (mg/mL) | 1.2^b^ (0.23) | 1.7^a^ (0.39) | 1.3^ab^ (0.25) | 1.3^ab^ (0.36) | 0.690 | 0.017 | | 0.040 |
| mIgM (µg/mL) | 9.7^b^ (2.81) | 9.5^b^ (2.85) | 9.8^b^ (2.47) | 19.3^a^ (2.87) | 0.006 | 0.018 | | 0.004 |
| ChT (mU/mL) | 331^a^ (41.7) | 234^b^ (27.7) | 266^ab^ (23.0) | 289^ab^ (17.3) | 0.531 | 0.013 | | 0.005 |

Values with different superscripts in same row (^a, b, c^) differ significantly (P<0.05) among treatments according to the BH procedure ([Benjamini and Hochberg, 1995](file:///E:/Doctorado/Experimental%20Early%20life%20programming/Papers/2nd%20paper/Table_Results.docx#_ENREF_5)).

^1^ % based on the total cells counted.

Abbreviations: BW: body weight; ADG: average daily gain; VFI: voluntary feed intake; FCE: feed conversion efficiency (kg feed/kg growth); WBC: white blood cells; Ig: immunoglobulin; s: serum; m: mucosal (saliva); ChT: chitotriosidase activity.

Table S11. Mean (standard deviation) of the Spearman correlation coefficient, relating the ruminal core bacterial taxa observed in 80% of goat kids (at genus level) with the average daily gain and feed efficiency (ADG and FCE, respectively) of goat kids at 4, 8, 14 and 20 weeks of age.

| Taxa | 4 weeks | | 8 weeks | | 14 weeks | | 20 weeks | |
| --- | --- | --- | --- | --- | --- | --- | --- | --- |
|  | AGD | FCE | AGD | FCE | AGD | FCE | AGD | FCE |
| **Bacteroidetes** |  |  |  |  |  |  |  |  |
| Uncul_Bacteroidales |  |  |  |  |  |  | -0.44ǂ | 0.52§ |
| BS11 |  |  |  |  |  | -0.63† |  |  |
| **Firmicutes** |  |  |  |  |  |  |  |  |
| Uncul_Clostridiales |  |  |  |  |  |  | 0.54§ | -0.42ǂ |
| *Mogibacterium* |  |  |  | -0.44ǂ |  |  |  |  |
| *Clostridium* |  |  |  |  |  |  | 0.44ǂ |  |
| *Coprococcus* |  | -0.41ǂ |  |  | -0.59§ | 0.42ǂ | -0.54§ |  |
| *Pseudobutyrivibrio* |  |  |  |  | -0.43ǂ |  | -0.41ǂ |  |
| Uncul_Ruminococcaceae |  |  |  |  |  | 0.58§ | 0.69† |  |
| Other_Ruminococcaceae |  |  | -0.46ǂ | 0.44ǂ |  |  |  |  |
| **Proteobacteria (***Desulfovibrio**) |  |  |  |  |  | 0.69† |  |  |

Significances are based on Spearman *P*-values: † = P < 0.01; § = P < 0.05; ǂ = 0.05 ≤ P < 0.10; gray fields, P ≥ 0.10.

* In the current microbial dataset, only one genus was detected within the Phyla Protobacteria (*Desulfovibrio*.

Table S12. Effect of prenatal (*L. leucocephala*) treatment (last 7 weeks of pregnancy) on white blood cell count and immune indices in colostrum of goats (n = 5).

| Index | Treatments | | | | SEM | | *P*-value |
| --- | --- | --- | --- | --- | --- | --- | --- |
|  | D+ | | D- | |  |  |  |
|  |  |  | |  | |  | |
|  |  | Blood | |  | |  | |
| Total WBC (*10^9^/L) | 11.8 | | 11.7 | | 0.28 | | 0.972 |
| Lymphocytes (%^1^) | 30.2 | | 28.4 | | 1.22 | | 0.483 |
| Neutrophils (%) | 69.6 | | 68.0 | | 2.08 | | 0.710 |
| Eosinophils (%) | 0.2 | | 3.4 | | 1.47 | | 0.307 |
| Monocytes (%) | nd | | nd | | - | | - |
|  |  | Colostrum | |  | |  | |
| ChT (U/mL) | 0.11 | | 0.06 | | 0.01 | | 0.093 |
| IgG (mg/mL) | 60.1 | | 32.8 | | 2.92 | | 0.002 |
| IgM (mg/mL) | 2.69 | | 2.37 | | 0.06 | | 0.034 |

Abbreviations: SEM: Standard error of means; WBC: white blood cells; ChT: chitotriosidase activity; Ig: immunoglobulin; nd: Not detected

^1^ % based on the total blood cells counted.

Figure S1. Principal coordinate analysis (PCoA) plot of bacterial 16S rRNA gene distribution at OTU level in rumen inocula collected from goat kids at 4 weeks (■), 8 weeks (●), 14 weeks (►) and 20 weeks (▲). PCoA plot is based on Bray-Curtis distances.

Figure S2. Relative abundance (%) of core successional microbiome (present in 80% of all animals) at the level of phyla (A) and genera (B) of goat kids at different ages. Kids were either treated (D+) or not (D-) with *L. leucocephala* forage meal prenatally (last 7 weeks of the pregnancy) and/or treated or not with yeast postnatally (birth to 2 month old, K+ vs. K-).

Figure S1. JPEG


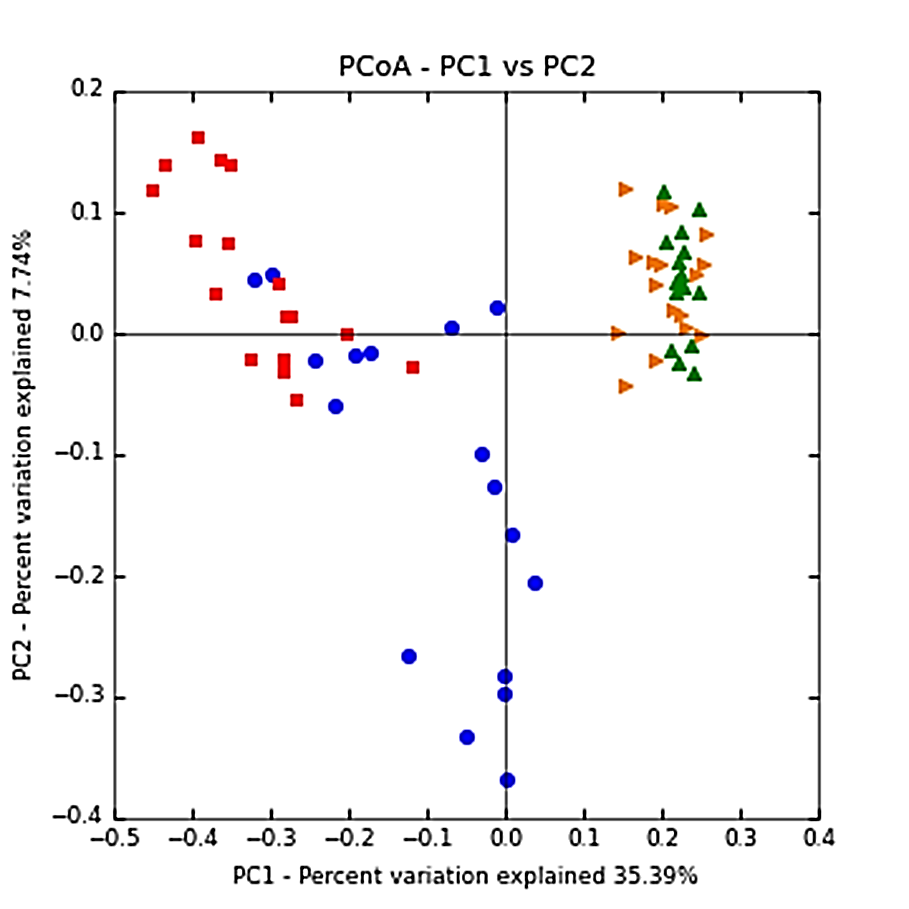


Figure S2. JPEG

**
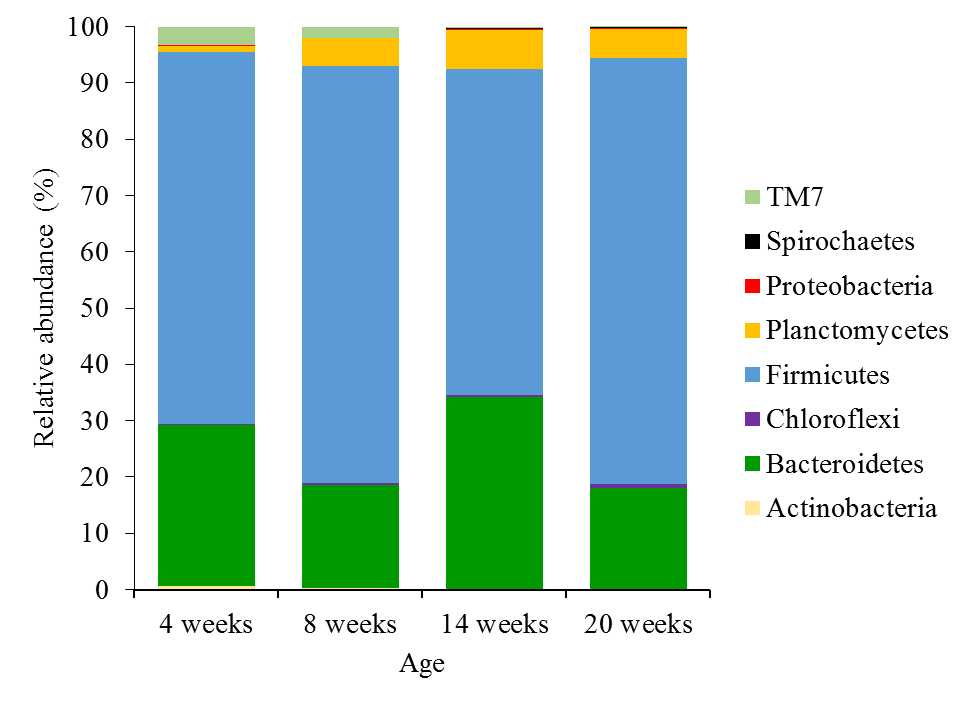
 A**

**
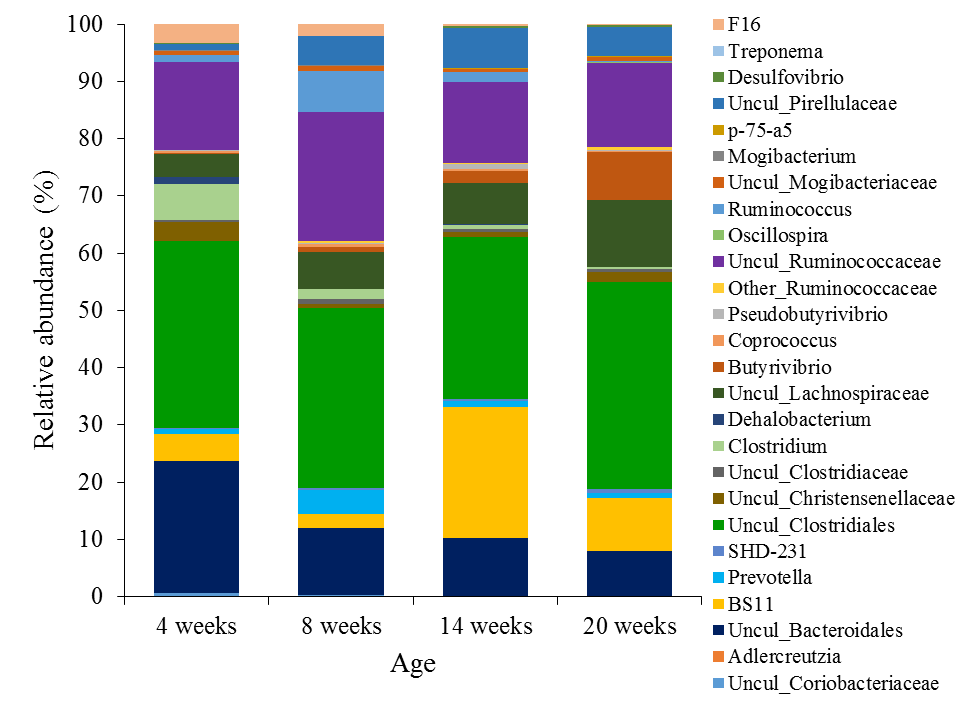
 B**
